# Photocleavable Mass-Tagged Oligonucleotide Probes for Multiplexed and Multiomic Tissue Imaging of Targeted Transcripts

**DOI:** 10.1021/jasms.5c00057

**Published:** 2025-07-11

**Authors:** Jonathan M. Bell, Gargey Yagnik, Leonardo G. Dettori, Philip Carvalho, Zhi Wan, Kenneth J. Rothschild, Mark J. Lim

**Affiliations:** † AmberGen, Inc., 44 Manning Road, Billerica, Massachusetts 01821, United States; ‡ Department of Physics and Photonics Center, Boston University, Boston, Massachusetts 02215, United States

**Keywords:** matrix-assisted laser desorption/ionization mass spectrometry, mass spectrometric imaging, photocleavable mass-tags, RNA, *in situ* hybridization, fluorescence *in situ* hybridization, spatial transcriptomics, spatial biology, multiplex imaging, spatial multiomics, immunohistochemistry

## Abstract

Many fluorescence-based *in situ* hybridization (FISH) methods have been developed to spatially resolve DNA (genes) and RNA (transcripts) in tissues. Signal amplification is achieved in a variety of ways, including branched DNA (bDNA) methods that create multiple fluorescent probe binding sites on the target nucleic acid. To avoid spectral overlap, high levels of multiplexing are achieved by extensive cycling, using a few nonoverlapping fluorophores per cycle. However, these methods can be slow, cause accumulating tissue damage, and are negatively impacted by autofluorescence. In addition, FISH-based methods alone do not provide a comprehensive multiomic picture of the complex biological contributions from the different molecular species in a tissue, including metabolites, nucleic acids, proteins, and xenobiotics. We report the development of novel photocleavable mass-tagged oligonucleotide probes generated by copper-free Click chemistry for use with amplified and multiplexed MALDI mass spectrometric imaging-based *in situ* hybridization (MALDI-ISH). These probes were successfully substituted for fluorescent detector probes using RNAscope but required no cycling. We also demonstrate a fully mass spectrometric workflow that enables multiomic imaging of label-free metabolites (lipids) and targeted transcripts from a single Alzheimer’s mouse brain tissue section. Furthermore, we demonstrate a triomic workflow where, in addition to label-free lipids, adding MALDI-ISH combined with MALDI-immunohistochemistry (MALDI-IHC) enables imaging of targeted transcripts and proteins on the same tissue section. K-means cluster analysis of multiomic biomarkers reveals spatial correlations of these various molecular species with Alzheimer’s plaques.

## Graphical abstract

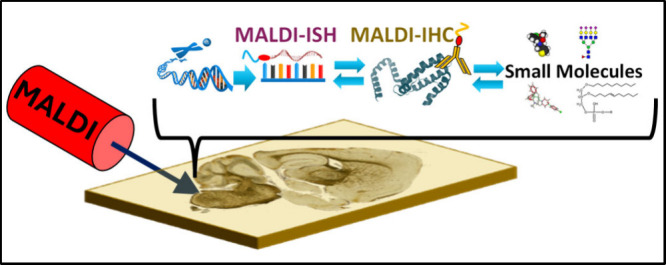

## Introduction

Spatially resolved transcriptomics of tissues, named “Method of the Year 2020” by Nature Methods, spans a range of approaches including fluorescence- and sequencing-based readouts.^1^ In the case of fluorescence, often termed fluorescence *in situ* hybridization (FISH),^2,3^ optical microscopy is used to detect the location of oligonucleotide probes. These probes bind to nucleic acid targets in the tissue and are optically coded by the presence of different fluorophores. In order to achieve high multiplexing, cyclic FISH methods have been introduced which read a few fluorophore colors at a time but perform many cycles^4–6^ and in some cases use barcoding such as MERFISH.^7^ These cyclic approaches as well as sequencing-based methods have pushed the number of transcripts that can be spatially profiled in a tissue to the thousands. However, these methods still do not provide a comprehensive multiomic approach on the same sample using the same instrument platform and therefore lack the ability to image more than just one biomarker class, such as nucleic acids. Yet, spatial multiomics is necessary to gain a comprehensive “holistic” view of the tissue biology from the same cell population within the tissue. This goal is difficult to achieve using serial tissue sections and/or different instrument platforms for the different “omic” imaging layers, which are difficult to align and undergo different sample processing.

Mass spectrometric imaging (MSI) in some respects is an ideal platform on which to build a multiomic spatial biology approach, owing to its unique capability to image the spatial distribution in tissues of a wide range of label-free small biomolecules such as metabolites (*e.g.*, lipids and glycans) and even xenobiotics such as drugs.^8–11^ MSI also provides inherently high multiplexing capability due to the very high mass accuracy, with mass error often less than a few ppm in the low mass range, and high mass resolution (*e.g.*, often >40,000 [Mass/fwhm]). This approach has been particularly successful using matrix-assisted laser desorption/ionization (MALDI) mass spectrometry (MS).^12–15^ Tentative analyte identification is based on high mass precision and resolution, with verification typically by tandem MS/MS fragmentation techniques.

While MSI of macromolecules such as proteins (*e.g.*, >25 kDa) has been achieved using both top-down and bottom-up approaches (*e.g.*, intact protein MSI such as in ref 16 and *in situ* proteolysis-based methods such as in ref 17), these methods have limitations. For instance, with bottom-up protein MSI, challenges include the competing goals of avoiding analyte spatial delocalization while achieving comprehensive liquid-phase *in situ* proteolysis. Moreover, the resultant highly complex mixtures of biomolecules at each “pixel” lead to ion suppression effects and, ultimately, lessened sensitivity and in many cases detection of only the most highly abundant species.^16,18^ In the case of nucleic acids, direct MSI of these macromolecules in tissues is impeded by several challenges such as low abundance, high mass, propensity to form salt adducts, and instability in the gas phase.^19^ Due to these challenges, several groups have developed targeted approaches based on the binding of affinity probes (*e.g.*, antibodies and oligonucleotides) to the macromolecular targets in the tissue and indirect detection of photocleavable mass-tags (PCMTs) conjugated to these probes. The photoreleased mass-tag, which is designed to have a different mass for each conjugated probe, serves as a type of code for the targeted macromolecule (Figure 1). These targeted approaches include trityl-based PCMTs attached to antibodies used in the TAMSIM method reported by Thiery et al.^20,21^ and photocleavable peptide mass-tags attached to antibody and oligonucleotide probes in the Tag-Mass method reported by Lemaire et al.^19^ In 1999, Olejnik et al.^22^ reported oligonucleotide probes conjugated to peptide-based PCMTs, which were hybridized to nucleic acid targets immobilized on glass bead resins, followed by nonimaging MALDI-MS detection of the photocleaved reporter ions. This work was based on a photocleavable linker (PC-Linker) containing a 1-(2-nitrophenyl)-ethyl photocleavable nucleus (PC-Nucleus), which exhibits a fast and highly efficient photoreaction.^23–25^ In 2021, we reported improvements on this chemistry and new workflows for the sensitive, highly multiplexed, and multiomic MALDI-MSI of targeted proteins in tissues based on PCMT-conjugated antibodies, termed MALDI-IHC.^26,27^

**Figure 1:**
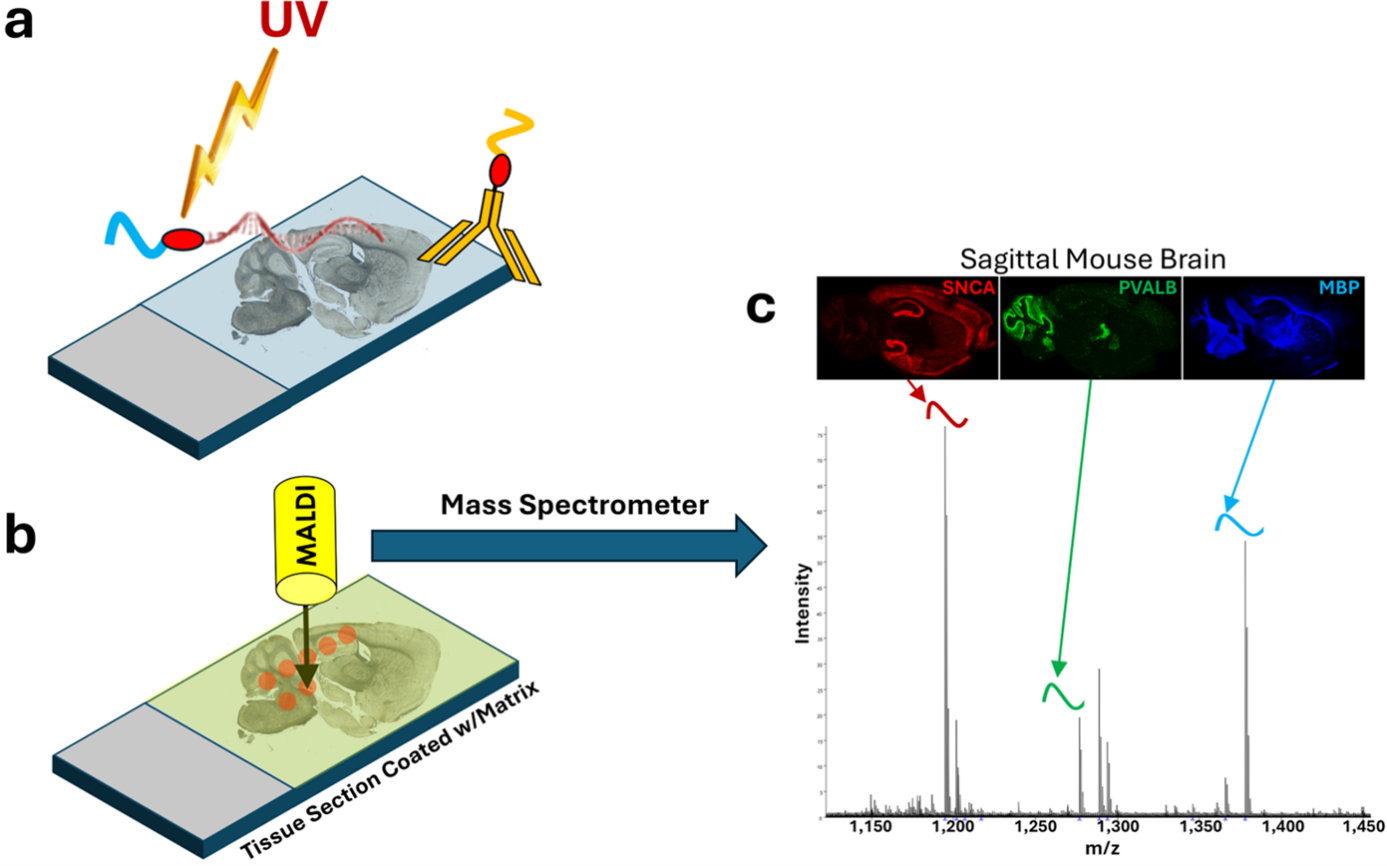
Basic features of MALDI-ISH and MALDI-IHC. (a) Tissue sections are stained with PCMT-conjugated affinity probes. In the case of MALDI-ISH, these consist of mass reporters (blue ribbon) conjugated to oligonucleotide hybridization probes (single-stranded DNA) through photocleavable linkers (red oval) targeting specific sequences of DNA or RNA in the tissue. Similarly, for MALDI-IHC, PCMTs (gold ribbon and red oval) are conjugated to antibodies targeting specific proteins. After staining but before matrix deposition, the tissue is exposed to UV light (lightning bolt) under dry conditions, which photoreleases the mass reporters (blue and gold ribbons) without delocalization. (b) The tissue section is then coated with the matrix, and the laser beam from the MALDI mass spectrometer scans individual spots on the tissue and records a mass spectrum at each spot (at each “pixel”). (c) Peak intensities at the *m*/*z* corresponding to different mass reporters (colored ribbons) can be used to reconstruct the 2D distribution of the corresponding targeted analytes, thereby creating a 2D image for each analyte. In the hypothetical example shown here, mass reporters and the resultant images for the targeted mRNA transcripts SNCA (α-synuclein; red), PVALB (parvalbumin; green), and MBP (myelin basic protein; blue) in a sagittal mouse brain tissue section are shown.

Here we report the adaptation of our prior antibody-based PCMTs for use with MALDI mass spectrometric imaging-based *in situ* hybridization (MALDI-ISH) for amplified spatial transcriptomics and spatial multiomics on the same tissue section using the same MALDI-MSI platform.^28^ These PCMTs utilize the core PC-Linker as previously reported for MALDI-IHC^26^ but modified to enable copper-free Click chemistry^29–31^ conjugation of the PCMT to the oligonucleotide detector probe. Importantly, the probes retain the advantages of the core PC-Linker used by MALDI-IHC. We demonstrate that these probes can be used in conjunction with a FISH-based multistage amplification method known as RNAscope.^32^ In this case, the PCMT-oligonucleotide probe serves as the final hybridization step and readout for the multistep amplified RNA detection instead of using probes with multiple fluorescence colors, which require cycling for more than 4 targets. We further demonstrate that these approaches are compatible with MALDI-based multiomic imaging workflows on fresh frozen (FF) tissue sections, where hundreds of untargeted metabolites (lipids), as well as several MALDI-ISH-targeted transcripts (and MALDI-IHC-targeted proteins), were spatially resolved on a single tissue section using the same MALDI-MS instrument. To demonstrate the utility of this approach, it was applied to tissue sections from hAbeta^SAA^ transgenic Alzheimer’s disease (AD) mouse brains, followed by K-means clustering analysis to show multiomic biomarker association with histological features such as the amyloid plaques.

## Methods

### Materials

Xylene (semiconductor grade), water (LC/MS grade), and methanol (LCMS grade) were from Alfa Aesar (Haverhill, MA). Acetone (HPLC grade) and SuperFrost Plus microscopy slides were from Fisher Scientific (Hampton, NH). Ethanol (bioreagent for molecular biology), *N*,*N* dimethylformamide (DMF, anhydrous, ≥99.8%), 1,5-diaminonaphthalene (DAN, 97%), sodium chloride (BioXtra, ≥99.5%), ammonium bicarbonate (BioUltra, ≥99.5%), phosphate buffered saline (PBS) (BioPerformance Certified, pH 7.4, P5368), octyl β-d-glucopyranoside (OBG) (50% [w/v] stock solution), α-cyano-4-hydroxycinnamic acid (CHCA), ammonium phosphate, and the ProteoMass Angiotensin II MALDI-MS Standards were from Sigma-Aldrich (St. Louis, MO). Tris HCl and Tris base (molecular biology grade), 0.5 M EDTA (pH 8; molecular biology grade), and molecular biology grade nuclease-free water were from Promega (Madison, WI). IntelliSlides were from Bruker Daltonics (Billerica, MA). Transgenic hAbeta^SAA^ (APP-SAA) FFPE and fresh frozen (FF) (embedded in 2% w/v CMC) mouse brain blocks were from The Jackson Laboratory (Bar Harbor, ME). C57 FFPE and FF (embedded in 2% w/v CMC) mouse brain blocks were from Zyagen (San Diego, CA). FFPE and FF tissue blocks were microtome sectioned (5 μm thickness) or cryosectioned (10 μm thickness) and mounted on slides by Zyagen (San Diego, CA). NAP-5 Sephadex G-25 Columns were from Cytiva (Marlborough, MA). LCMS grade trifluoroacetic acid (TFA), SSC buffer 20× (molecular biology grade), Bond-Breaker TCEP solution, sodium acetate solution (3 M, pH 5.2), EZ-Link Maleimide-PEG4-DBCO, and LCMS grade acetonitrile were from Thermo Scientific (Waltham, MA). RNAscope HiPlex metal-ready probes T1-T12, mouse-directed transcript-specific RNAscope HiPlex Probes (Z-probes), RNAscope Probe Diluent, PretreatPRO solution and the RNAscope Intro Pack for HiPlex Reagents Kit (Catalog 324442) were from Bio-Techne/Advanced Cell Diagnostics (ACD) (Newark, CA). Paraformaldehyde 16% Aqueous Solution EM grade was from Electron Microscopy Sciences (Hatfield, PA). The H&E Staining Kit (ab245880) was obtained from ABCAM (Cambridge, MA). The MALDI-IHC antibody probes were from AmberGen Inc. (Billerica, MA), see Supplementary Table S1 for clones and PCMT assignments.

### PCMT Conjugation to Oligonucleotide Detector Probes

Molecular biology grade water (MBG-Water; see Materials) was used for all reagents in the Methods section unless otherwise specified. As the starting oligonucleotide material for labeling with PCMTs, RNAscope HiPlex metal-ready probes were purchased from Bio-Techne/Advanced Cell Diagnostics (ACD) (Newark, CA), which corresponded to the T1-T12 detector oligonucleotides for 12-plex RNAscope assays but without fluorescent labels. These “label-ready” oligonucleotide probes contain a disulfide group which, when reduced, results in a terminal thiol/sulfhydryl group. These lyophilized oligonucleotides were resuspended in 80 μL of MBG-Water to a final concentration of 250 μM in 1.5 mL microcentrifuge tubes. Concentration was confirmed by absorbance at 260 nm in a Nanodrop One^C^ microvolume spectrophotometer (Thermo Scientific, Waltham, MA). A 50 μL portion of the 250 μM oligonucleotides was mixed with 5 μL of 0.5 M Bond-Breaker TCEP solution and reacted for 30 min with gentle mixing to reduce the disulfide group on the oligonucleotides and generate a terminal thiol/sulfhydryl group.

Ethanol precipitation was next performed to purify the oligonucleotides as follows: 5.5 μL of 3 M sodium acetate (final concentration 0.3 M) was added to the oligonucleotides and mixed briefly by vortexing. 150 μL (2.5 volumes) of 100% EtOH (prechilled to −20 °C) was then added and mixed briefly by vortexing. The solutions were placed into a −20 °C freezer for at least 30 min. The oligonucleotides were spun down for at least 30 min at maximum speed in a standard refrigerated microcentrifuge (∼15,000*g*). The supernatant was then removed, being careful not to disturb the oligonucleotide pellet. 500 μL of ice-cold 70% ethanol was gently added to wash the pellet, and the centrifugation was repeated for only 3 min. The supernatant was again removed, being careful not to disturb the pellet. The tubes were then left with the lids open to briefly air-dry the pellets (1–3 min).

Oligonucleotide pellets were then resuspended in 100 μL of PBS, and the concentration was determined by absorbance at 260 nm on a Nanodrop One^C^ microvolume spectrophotometer. Based on this measurement, the oligonucleotides were diluted to 50 μM in PBS. 3.9 mM EZ-Link Maleimide-PEG4-DBCO was freshly prepared by dissolving 1 mg (MW 647.74) in 400 μL of anhydrous DMF. 52 μL of the 3.9 mM EZ-Link Maleimide-PEG4-DBCO solution (200 nmoles) was added to the 50 μM oligonucleotide solutions followed by mixing. The reaction was carried out for 2 h with gentle mixing. The resultant products are referred to as the DBCO modified oligonucleotide probes.

PCMT labeling of the DBCO-modified oligonucleotide probes was performed next using copper-free Click chemistry.^29–31^ The peptide-based azide-activated PCMT oligonucleotide labeling reagent (PCMT-azide, see Figure 2a) was synthesized in the same manner as done previously for *N*-hydroxysuccinimide (NHS) activated PCMT antibody labeling reagents, which is based on standard Fmoc-mediated solid-phase peptide synthesis (SPPS).^33^ However, instead of generating an NHS-ester on the ε-amine of the lysine (K) side chain in the PCMT labeling reagent, an azido-lysine was created (see Figure 2a for the Lysine [K] position in the PCMT). This was accomplished using Fmoc-azidolysine in the SPPS process that was used to create the PCMTs. To perform oligonucleotide labeling using copper-free Click chemistry, a 10 mM stock of PCMT-azide reagent was prepared in anhydrous DMF. 30 μL of the 10 mM PCMT-azide (300 nmoles) was then added to the DBCO modified oligonucleotide probes. The reaction was performed overnight protected from light and with gentle mixing.

**Figure 2:**
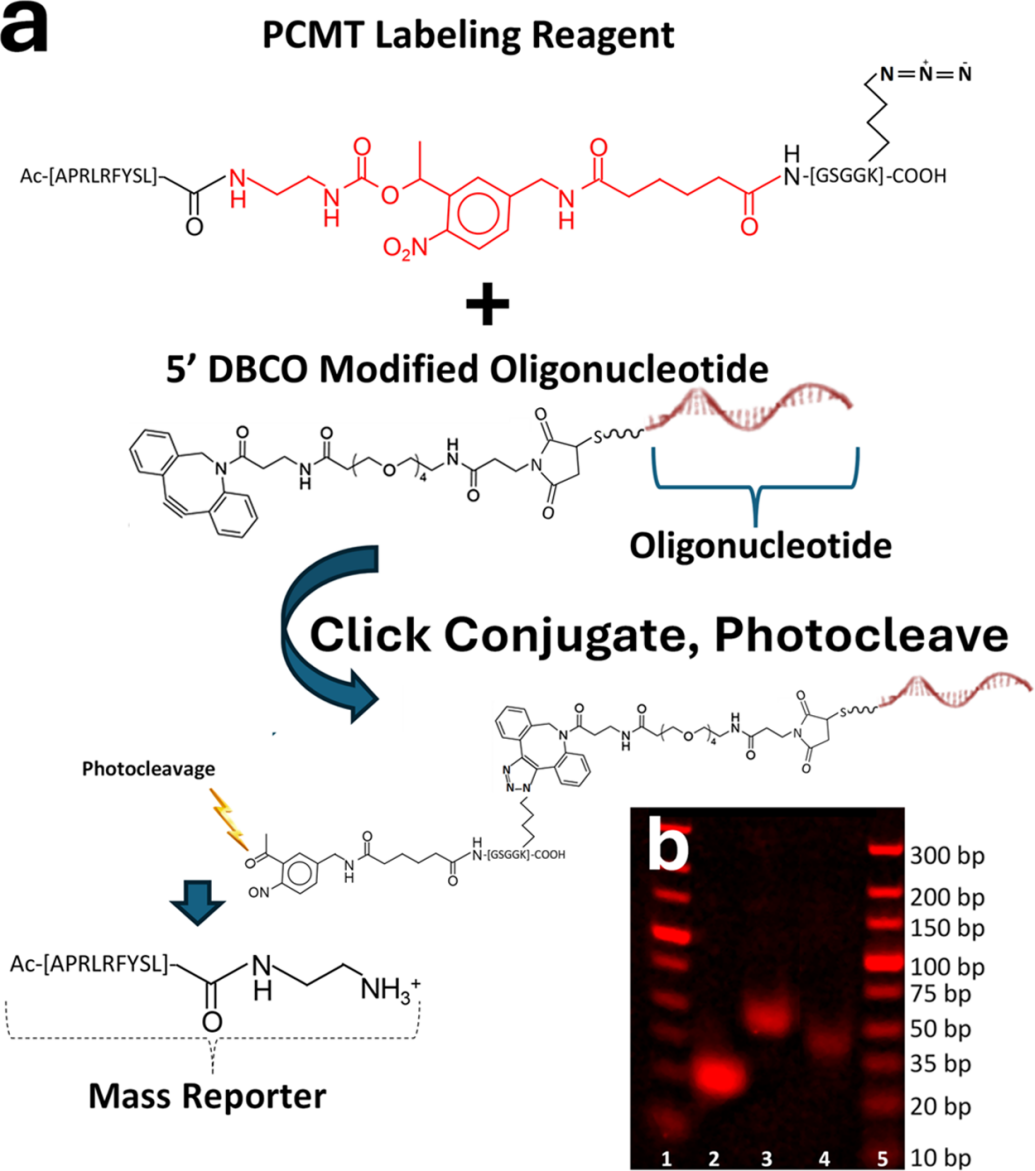
Structure of the azide-activated PCMT labeling reagent, as well as chemical synthesis and verification of the PCMT-conjugated oligonucleotide probes (PCMT-oligonucleotides). (a) The PCMT labeling reagent is produced by standard Fmoc-based solid-phase peptide synthesis (SPPS) using a custom-designed photocleavable amino acid analogue to introduce the photocleavable linker (PC-Linker; red structures). The PCMT labeling reagent has an azide moiety near the C-terminus introduced during SPPS using Fmoc-azidolysine. The PCMT labeling reagent is conjugated to DBCO (alkyne) modified oligonucleotides using copper-free Click chemistry. Following photocleavage (lightning bolt), the mass reporter, which comprises a variable amino acid sequence and a portion of the PC-Linker, is released, which generates a terminal primary amine group after photocleavage to assist with positive ion mode MALDI-MS (example mass reporter shown using single letter code; note the peptide is N-terminal acetylated as denoted by the “Ac”). (b) PCMT-oligonucleotides are verified by agarose gel electrophoresis, with an example shown here. Lanes 1 and 5 are the molecular weight ladders (standards), lane 2 is the unmodified source oligonucleotide (lacking the DBCO and PCMT modifications), lane 3 is the fully intact PCMT-oligonucleotide, and lane 4 is the photocleaved PCMT-oligonucleotide (oligonucleotide portion, which retains a portion of the photocleaved PC-Linker and the DBCO linker).

Removal of unreacted PCMT-azide and buffer exchange was then achieved using NAP-5 G-25 Sephadex columns per the manufacturer’s instructions against TE-150 mM NaCl buffer (TE [10 mM Tris, pH 8.0, 1 mM EDTA] with 150 mM NaCl). Briefly, the column top and bottom caps were removed, and the storage buffer allowed to flow through to waste. The columns were then pre-equilibrated with 3 full column fillings of TE-150 mM NaCl (flow-through to waste). This was ∼10 mL of total pre-equilibration buffer volume. When the last of the pre-equilibration buffer had fully entered the column, the oligonucleotide samples were added, and the flow-through was discarded to waste. After the sample had fully entered the column, 190 μL (or enough to total 500 μL loaded) of TE-150 mM NaCl was added, and the flow-through again was discarded to waste. Finally, after the added TE-150 mM NaCl had fully entered the column, the sample was eluted with ∼0.7 mL of TE-150 mM NaCl, with the flow-through (eluate) collected this time into a clean 1.5 mL microcentrifuge tube. The oligonucleotide concentration was determined by absorbance at 260 nm on a Nanodrop One^C^ microvolume spectrophotometer.

The PCMT-conjugated oligonucleotide probes were further purified by ethanol precipitation in batch sizes of 300 μL of the aforementioned NAP-5 eluate as follows: 33.33 μL of 3 M sodium acetate (final concentration 0.3 M) was added to the oligonucleotides and mixed briefly by vortexing. 840 μL (2.5 volumes) of 100% EtOH (prechilled to −20 °C) was then added and mixed briefly by vortexing. The solutions were placed into a −20 °C freezer for at least 30 min. The oligonucleotides were spun down for at least 30 min at maximum speed in a standard refrigerated microcentrifuge (∼15,000*g*). The supernatant was then removed, being careful not to disturb the oligonucleotide pellet. 500 μL of ice-cold 70% ethanol was gently added to wash the pellet, and the centrifugation was repeated but only for 3 min. The supernatant was again removed, being careful not to disturb the pellet. The tubes were then left with the lids open to briefly air-dry the pellets (1–3 min). The final PCMT-conjugated oligonucleotide probes (PCMT-oligonucleotides) were resuspended in 100 μL of TE Buffer (10 mM Tris, pH 8.0, 1 mM EDTA), and the oligonucleotide concentration was determined by absorbance at 260 nm on a Nanodrop One^C^ microvolume spectrophotometer.

Conjugation of the PCMTs to the oligonucleotides was confirmed using the E-Gel Power Snap Electrophoresis System using precast E-gels with SYBR Safe DNA stain according to the manufacturer’s instructions (Thermo Scientific, Waltham, MA).

### Amplified MALDI-ISH on Fresh Frozen Tissue Sections Using the HiPlex RNAscope Assay

The HiPlex RNAscope assay was essentially performed according to the manufacturer’s instructions for fresh frozen (FF) tissue sections (Bio-Techne/Advanced Cell Diagnostics [ACD], Newark, CA), except the PCMT-oligonucleotides described above were used instead of fluorescent oligonucleotides at the detector probe step and the final washes were modified to remove salts that are incompatible with MALDI-MSI. Note that reagents used from the commercial RNAscope Intro Pack for HiPlex Reagents Kit (see Materials) are indicated by an asterisk (*) in all procedures detailed in the remainder of the Methods section.

Transgenic Alzheimer’s hAbeta^SAA^ (APP-SAA) and wild-type (WT) C57 FF mouse brain tissue sections (see Materials) were used as the samples, and the 10 μm cryosections were mounted on Bruker IntelliSlides (see Materials). FF tissue slides were removed from −80 °C storage and immediately immersed in a 4% PFA fixative (prepared by dilution of a 16% commercial PFA stock [see Materials] in PBS) in a polypropylene Coplin jar for 60 min. Unless otherwise noted, all bulk volume steps were in Coplin jars with an excess of solution (∼70 mL). Slides were washed two times for 2 min each in PBS. Tissues were then dehydrated with an aqueous/ethanol series as follows (5 min each): 50% ethanol (v/v in MBG-Water), 70% ethanol, and 2× with 100% ethanol. Slides were air-dried for 5 min at room temperature.

Tissue sections were then each surrounded by hydrophobic barrier pen markings to facilitate the following small volume reagent incubations (*i.e.*, 100 μL/cm^2^; not performed in Coplin jars unless otherwise noted): Protease IV* prewarmed to room temperature was incubated with the tissue for 30 min at 40 °C in a humidified chamber. Slides were rinsed 2× briefly with PBS (∼70 mL in Coplin Jar). The mixture of mouse-directed transcript-specific Z probes (see Materials), each diluted 1:50 in RNAscope Probe Diluent, was applied to one of the tissue sections on the slide. To a second serial tissue section on the slide a negative control mixture of Z-probes*, diluted in the same manner, was added (these were Z-probes targeting unrelated bacterial transcripts absent from the mouse brain tissue sample, but comprising the same amplification tails). Slides were incubated in a humidified chamber for 2 h at 40 °C to allow for Z-probe hybridization to their targets.

The following steps used 10 cm glass Petri dishes and ∼20 mL of solution per step. Note that where 1× Wash Buffer is indicated in the Methods section, it was prepared by dilution of 50× Wash Buffer* in MBG-Water. Slides were washed with gentle mixing 2× for 2 min each with 1× Wash Buffer. Slides were stored overnight in 5× SSC (750 mM NaCl and 75 mM sodium citrate, pH 7.0). The next day, slides were washed 2× for 2 min each with 1× Wash Buffer.

The following small-volume reagent incubations were performed within the hydrophobic barrier pen markings (*i.e.*, 100 μL/cm^2^ unless otherwise noted): Slides were incubated at 40 °C for 30 min with HiPlex Amp 1* in a humidified chamber. Slides were then washed 2× for 2 min with 1× Wash Buffer (∼20 mL in Petri dish). Slides were incubated at 40 °C for 30 min with HiPlex Amp 2* in a humidified chamber. Slides were then washed 2× for 2 min with 1× Wash Buffer (∼20 mL in Petri dish). Slides were incubated at 40 °C for 30 min with HiPlex Amp 3* in a humidified chamber. Slides were then washed 2× for 2 min with 1× Wash Buffer (∼20 mL in Petri dish). Slides were incubated at 40 °C for 30 min with a mixture of the PCMT-oligonucleotides (20 nM each diluted in RNAscope Probe Diluent) in a humidified chamber. Slides were then washed 2× for 2 min with 1X Wash Buffer (∼20 mL in Petri dish). The final washes to remove salts that are incompatible with MALDI-MSI were as follows: Slides were rinsed briefly for 10 s and then 3× for 2 min, with excess 50 mM ammonium bicarbonate at each step, in a 10 cm glass Petri dish with gentle shaking. Slides were then dried for 1.5 h in a vacuum desiccation chamber.

### Multiomic Workflows Using MALDI-ISH for Imaging Untargeted Lipids as Well as Targeted RNAs on the Same Tissue Section

Unprocessed FF tissue sections were first directly subjected to MALDI-MSI of untargeted, endogenous, and label-free lipids. This lipid imaging was performed as previously described using sublimation/recrystallization of a 1,5-diaminonaphthalene (DAN) matrix and negative ion mode MALDI-MSI,^33^ in this case on a Bruker timsTOF fleX MALDI-2 (Bruker Daltonics, Billerica, MA) system. Following MALDI-MSI of lipids, the remaining DAN matrix was removed, and the tissue was simultaneously fixed by washing 2× with −80 °C acetone for 3 min each in Coplin jars (∼70 mL), followed by drying the slides for 10 min in a vacuum desiccator. In cases where only MALDI-ISH was performed after MALDI-MSI of lipids, MALDI-ISH was performed as described earlier in the Methods section starting with the 4% PFA fixation. In cases where both MALDI-ISH and MALDI-IHC were performed after MALDI-MSI of lipids, MALDI-ISH was performed as described earlier in the Methods section, with the following exceptions: The steps of surrounding the tissue sections with a hydrophobic barrier pen and Protease IV treatment as described earlier in the Methods section were replaced with treating the tissue slides with 1× Target Retrieval Solution* at 96 °C for 30 min using a capped polypropylene Coplin jar (∼70 mL). Tissue slides were then rinsed briefly with MBG-Water and washed with 100% ethanol for 3 min using 10 cm glass Petri dishes and ∼20 mL of solution per step. Tissue slides were then air-dried at room temperature for 5 min. The hydrophobic barrier pen was then applied around the tissue sections to facilitate a small volume incubation (*i.e.*, ∼100 μL/cm^2^ in a humidified chamber) with the PretreatPRO solution (see Materials), which was prewarmed to room temperature and incubated on the tissue sections in the humidified chamber at 40 °C for 30 min. The remaining MALDI-ISH steps were performed as described earlier in the Methods section, starting after the Protease IV treatment (*i.e.*, the Protease IV* step was not performed in this multiomic workflow). Following the last wash with 1× Wash Buffer in the MALDI-ISH procedure (before the final washes in 50 mM ammonium bicarbonate of the MALDI-ISH procedure), a simplified MALDI-IHC procedure was next performed. Tissue slides were rinsed briefly for 10 s and then 3× for 2 min, with 1× TBS buffer at each step (50 mM Tris, pH 7.4, 200 mM NaCl), in a 10 cm glass Petri dish with gentle shaking. The MALDI-IHC PCMT-antibody probe mix (2.5 μg/mL each PCMT-antibody; see Supplementary Table S1 for PCMT-antibody list) was prepared in 1× TBS-OBG buffer (TBS with 0.05% [w/v] OBG), and tissue sections were incubated with the PCMT-antibody probe mix for 1 h at 37 °C inside a humidified chamber (*i.e.*, ∼100 μL/cm^2^ using the hydrophobic barrier pen markings for this low volume incubation). Tissue slides were rinsed briefly for 10 s and then 3× for 2 min, with 1× TBS buffer at each step (50 mM Tris, pH 7.4, 200 mM NaCl), in a 10 cm glass Petri dish with gentle shaking. To remove salts that are incompatible with MALDI-MSI, the tissue slides were next rinsed briefly for 10 s and then 3× for 2 min, with excess 50 mM ammonium bicarbonate at each step, in a 10 cm glass Petri dish with gentle shaking. Slides were then dried for 1.5 h in a vacuum desiccation chamber.

### MALDI-ISH on FFPE Tissue Sections

The HiPlex RNAscope assay was essentially performed according to the manufacturer’s instructions for formalin fixed paraffin embedded (FFPE) tissue sections (Bio-Techne/Advanced Cell Diagnostics [ACD], Newark, CA), except the PC-MT-oligonucleotides were used instead of fluorescent oligonucleotides at the detector probe step and the final washes were modified to remove salts that are incompatible with MALDI-MSI. The detailed protocol was as follows:

hAbeta^SAA^ (APP-SAA) FFPE mouse brain tissue sections (see Materials) were used as the sample, and the 3 μm microtome sections were mounted on Fisherbrand Superfrost Plus Microscope Slides. Before use, slides were heated for 1 h at 60 °C in a laboratory oven to promote tissue adhesion to the surface.

Unless otherwise noted, all bulk volume steps were in Coplin jars using an excess of solution (∼70 mL). Slides were incubated 2× for 5 min each in xylene and 2× for 2 min each in ethanol and then dried in a laboratory oven for 5 min at 60 °C.

Slides were then incubated for 15 min at 96 °C in 1× Target Retrieval Reagent* (performed using a Coplin jar placed in a water bath). Slides were then incubated for 15 s in MBG-Water, then 3 min in 100% ethanol, and dried in a laboratory oven for 5 min at 60 °C.

Tissue sections were then each surrounded by hydrophobic barrier pen markings (dried 10 min or more) to facilitate the following small-volume reagent incubations (*i.e.*, 100 μL/cm^2^; not performed in Coplin jars unless otherwise noted): Protease III*, prewarmed to room temperature, was incubated with the tissue for 30 min at 40 °C in a humidified chamber. Slides were rinsed 2× for 15s each with MBG-Water (∼70 mL in Coplin Jar).

The remaining steps are the same as performed with the standalone MALDI-ISH procedure (*i.e.*, not the multiomic procedure) detailed earlier in the Methods section (starting after the protease treatment and subsequent PBS washes detailed earlier).

### MALDI Mass Spectrometry Imaging (MALDI-MSI) of PCMTs

Following the sample preparation procedures for MALDI-ISH and the final slide drying as detailed earlier in the Methods section, PCMT photocleavage was performed on the dried slides as reported previously.^33^ The matrix was then applied using an HTX M3+ Sprayer (HTX Technologies, LLC, Chapel Hill, NC) according to the following parameters: nozzle temperature 60 °C, flow rate 0.1 mL/min, nozzle velocity 1350 mm/min, 8 passes, track spacing 3 mm, nozzle height 40 mm, pattern CC, PSI 10, gas flow 2 L/min and a dry time of 10 s. The base solvent was 70% acetonitrile, 0.1% TFA, and 10 mM ammonium phosphate, and the matrix was 10 mg/mL α-cyano-4-hydroxycinnamic acid (CHCA) in base solvent with a 2 nM final concentration of a ProteoMass Angiotensin II MALDI-MS Standard (see Materials). Following matrix application, slides were subjected to matrix recrystallization as reported previously.^33^

All MALDI-MSI measurements were performed on a timsTOF fleX MALDI-2 instrument (Bruker Daltonics, Billerica, MA) using the following parameters: reflector mode (positive ion mode for PCMTs and negative ion mode for direct lipid analyses); pixel size 20 μm with 16 μm beam scan continuous raster scanning; 10 kHz laser frequency; 100–300 laser shots/pixel; and 40–70% typical laser power setting. The MALDI-MSI signals were typically normalized using the root-mean-square (RMS) algorithm (in some cases left un-normalized where noted later in the Methods section). The tissue lipid and PCMT image generation, normalization, and spectral analyses were performed by using SCiLS Lab software (Bruker Daltonics, Billerica, MA). TIFF images of MALDI-MSI and MALDI-ISH data were exported from the SCiLS Lab and used to compose the figures without further modification other than resizing the image as needed and adding annotations such as arrows and circles to label features (image display settings used in SCiLS to generate the TIFFs are indicated in the figure legends).

### Post-MALDI H&E Staining

In some cases, conventional H&E (hematoxylin and eosin) staining was performed after MALDI-MSI of the PCMTs. The imaged slides were washed in a glass Petri dish using excess 100% methanol 3× for 2 min with gentle agitation to remove the MALDI-MSI matrix. Tissue slides were then rehydrated with excess reagent grade water for 10 min. Conventional H&E staining was performed according to the manufacturer’s instructions (ABCAM H&E Staining Kit ab245880), followed by brightfield microscopy on an Olympus VS200 whole slide imaging microscope with a 40x objective.

### Data Processing and Analysis

For all aforementioned MALDI-ISH experiments on FF and FFPE tissues, the transcript targets, oligonucleotide tail assignments, suppliers, and PCMT assignments for detector oligonucleotide probes are listed in Table 1. MALDI-MSI data were processed using Bruker’s SCiLS Lab Version 2025b Pro software. For positive-mode, targeted MALDI-ISH data the following process was used: (i) data were normalized using the RMS method; (ii) peaks corresponding to each reporter mass-tag were manually picked, and (iii) data were exported as OME-TIFF image files using the peak area as the interval processing mode and enabling hotspot removal. For negative-mode, untargeted lipid data the following process was used: (i) data were not normalized; (ii) peaks were automatically picked using the T-Rex^2^ (QTOF) algorithm with weak filtering, 100% coverage and relative intensity threshold of 2% resulting in 250 *m*/*z* values; (iii) the generated peak list was manually inspected to remove low quality peaks (*e.g.*, signal coming solely from outside the tissue section) resulting in 143 *m*/*z* values; (iv) in order to only maintain biologically relevant *m*/*z* values, the filtered list was compared against the LIPID MAPS Structure Database (LMSD) using the “bulk structure search” function from the Lipid Maps Web site (performed on 02/03/2025) with the options of [M – H]^−^ ion, all lipid classes, and ± 0.01 *m*/*z* mass tolerance resulting in 104 *m*/*z* values (tentative assignments can be seen in Supplementary Table S2);^34–37^ and (v) the peak list was imported back into SCiLS Lab and a final list including all 104 peaks was selected to be exported as an OME-TIFF image file using the peak area as the interval processing mode and enabling hotspot removal (out of all tentative assignments, only one lipid name was selected as a representative for each *m*/*z* value in the following analysis). After the 2 OME-TIFF images were exported from SCiLS Lab, they were coregistered using a custom workflow involving FIJI’s plugins “Register Virtual Stack Slices” and “Transfer Virtual Stack Slices” (FIJI version 2.14), which make use of the scale-invariant feature transform (SIFT) algorithm for feature detection for image registration.^38,39^ A Python (version 3.11.5) custom script using the PyIMageJ (version 1.4.1) and the wxPython (version 4.2.1) libraries was written to facilitate the coregistration workflow, which consisted of the following steps: (i) one image was selected from each OME-TIFF file to be used for the feature-based alignment, in this case, the MBP transcript from the MALDI-ISH data and *m*/*z* 822.5339 (tentatively assigned as PS 39:5) from the lipid data (ideally, these will be images of biomarkers which are known to colocalize or whose spatial distribution strongly resemble each other); (ii) “Register Virtual Stack Slices” calculated the transformations to align both data sets using a “Rigid” feature finding model and a “Rigid” registration model (rigid registration was selected since both data sets refer to the same tissue section); and (iii) “Transform Virtual Stack Slices” applied the transformations to the respective images without using interpolation to minimize image distortion.^40^ After the 114 TIFF images were aligned, a Region of Interest (ROI) was defined around the cerebral cortex for a K-means clustering analysis focused on this anatomical region of the mouse brain since it is known to be largely affected by the formation of amyloid plaques.^41–44^ The workflow was conducted using custom Python scripts to prepare the images for analysis, and the clustering was conducted using the Sklearn library. First, the signal intensity for each pixel was standardized on a biomarker basis to account for variations in measurement units and scales using the standard scaler function, followed by an implementation of the Lloyd’s K-means clustering algorithm^45,46^ using the KMeans function with the total number of clusters varying from 2 to 20 using default Sklearn parameters (algorithm was repeated 10 times with different centroid seeds per analysis and the best output in terms of inertia,^47–49^ or squared error, was selected). Based on the ability to identify an amyloid plaque cluster while still maintaining a low number of clusters, 13 clusters were chosen as the optimal number to analyze the spatial association of the 114 different biomarkers and a cluster centroid table (Supplementary Table S3) was generated to describe the contributions of the different biomarkers to each cluster, as well as a table listing the top 10 biomarkers defining each cluster (Supplementary Table S4).^47–49^ The K-means clustering analysis workflow was conducted using custom Python scripts, including the following packages/libraries: Sklearn (version 1.3.0), cv2 (version 4.9.0.80), NumPy (version 1.24.3), Matplotlib (version 3.7.2), Pandas (version 2.0.3), and Seaborn (version 0.12.2).

**Table 1:** PCMT-Oligonucleotides Used in MALDI-ISH Experiments on FF and FFPE Tissues^a^

Target (Oligonucleotide Tail)	Raw Material Supplier	PCMT ID	Monoisotopic *m*/*z*
GLUT1/SLC2A1 or CDH5 (T5)	Bio-Techne/ACD	PC-MT-14.03	1102.5792
APP (T6)	Bio-Techne/ACD	PC-MT-2.00	1194.6530
SNCA or MBP (T7)	Bio-Techne/ACD	PC-MT-7.11	1201.6760
CDH5 or SNCA (T2)	Bio-Techne/ACD	PC-MT-1.00	1206.7106
Tau/MAPT or TUBB3 (T12)	Bio-Techne/ACD	PC-MT-14.09	1276.6433
CTSD (T8)	Bio-Techne/ACD	PC-MT-7.13	1288.7080
GFAP (T4)	Bio-Techne/ACD	PC-MT-1.02	1293.7426
NEFH or Tau/MAPT (T10)	Bio-Techne/ACD	PC-MT-7.14	1345.7295
PVALB (T3)	Bio-Techne/ACD	PC-MT-2.05	1365.7174
MBP or NeuN (T11)	Bio-Techne/ACD	PC-MT-1.05	1377.7749

a ACD = Advanced Cell Diagnostics.

## Results and Discussion

### PCMT-Oligonucleotide Probe Design and Synthesis

Ideally, MALDI-ISH PCMT labeling reagents should facilitate efficient chemical conjugation to oligonucleotides and, after photocleavage by UV-illumination, release a single PCMT photoproduct, referred to as the mass reporter, instead of multiple photoproducts, which can complicate multiplex MALDI-MSI detection and decoding. For this purpose, we designed an azide-activated peptide-based PCMT labeling reagent (PCMT-azide) suitable for oligonucleotide conjugation by copper-free Click chemistry,^29–31^ which is similar to the design and synthesis of *N*-hydroxysuccinimide (NHS) activated PCMT antibody labeling reagents described previously for highly multiplex MALDI-IHC.^33^ However, instead of generating an NHS-ester on the ε-amine of the lysine (K) side chain in the PCMT labeling reagent, an azido-lysine was created. This was accomplished using Fmoc-azidolysine in standard Fmoc solid-phase peptide synthesis (SPPS) to create the azide moiety, which reacts by Click chemistry with alkynes present on modified oligonucleotides. Details of PCMT-azide conjugation to 5′ DBCO (alkyne) modified oligonucleotides (referred to as PCMT-oligonucleotides following conjugation) and the subsequent photocleavage reaction are shown in Figure 2a and described in detail in the Methods section. Importantly, the resulting mass reporter has a structure identical to that of the mass reporters used for MALDI-IHC, including a positively charged terminal amine group generated only upon photocleavage, to facilitate sensitive MALDI-MSI in the positive ion mode. It also lacks the photocleaved PC-Nucleus (*i.e.*, phenyl ring from the PC-Linker), which would limit the ability to perform sensitive and highly multiplex detection since the mass reporter would comprise multiple photoproducts.^50^

Figure 2b shows the results of agarose gel electrophoresis on one example PCMT-oligonucleotide, whereby the unconjugated source oligonucleotide runs at the expected position (∼20 bp, Lane 2). Following PCMT copper-free Click chemistry conjugation to the oligonucleotide and purification to remove unreacted PCMT (see Methods), a clear gel shift is observed (Lane 3), indicating the increase in mass (running approximately equivalent to 60 bp; note that with the peptide-based PCMT attached and having a different mass to charge ratio than the oligonucleotide component, the molecular weight of the conjugate in agarose gel electrophoresis cannot be readily interpolated from the calibration standards). The PCMT-oligonucleotide conjugate runs as a single species with no remaining unconjugated source oligonucleotide detected, indicating efficient PCMT conjugation. Following photocleavage of the PCMT-oligonucleotide, the mass is reduced (Lane 4). However, due to the intermediate Maleimide-PEG4-DBCO linker used in conjugation (see Methods), and remnants of the photocleaved PC-Linker that remain on the oligonucleotide (see Figure 2a, oligonucleotide conjugate after photocleavage), the mass is not fully reduced to the original unconjugated oligonucleotide. Instead, upon photocleavage, an intermediate mass between the original unconjugated source oligonucleotide and the intact PCMT-oligonucleotide is observed (running equivalent to ∼40 bp).

### Amplified and Multiplexed MALDI-ISH Imaging

Transcript signal amplification for MALDI-ISH was achieved using RNAscope technology,^32^ which is commercially available from Bio-Techne/Advanced Cell Diagnostics (ACD) (Newark, CA). PCMT-oligonucleotide detector probes were produced and substituted for the fluorescent oligonucleotide detector probes in a multistage RNAscope amplification scheme but without the need for cycling. RNAscope is an example of the more general method of branched DNA (bDNA) amplification.^51^ This involves the formation of a “tree” of branched DNA template sequences bound to the target transcript, creating multiple hybridization sites for the binding of labeled oligonucleotide detector probes and thereby providing multiple stages of amplification depending on the number of branching steps. In the case of RNAscope, as adapted here for MALDI-ISH, the amplification is depicted in Figure 3 and consists of the following: (i) Hybridization of two transcript-specific Z-probes, which bind in tandem to adjacent sequences on the target transcript and together act as a template for the preamplifier probe. The lower-region of each Z-probe is designed to recognize an 18–25-base transcript sequence (total of up to 50-mer sequence on the target transcript for the two tandem Z-probes). The top portion (referred to as a tail) of the two tandem Z-probes form a 28-base sequence, which acts as a template for the preamplifier oligonucleotide probes. As many as 20 tandem Z-probe pairs per transcript species are deployed to increase sensitivity and compensate for transcript degradation, which is common in FFPE tissue specimens, especially when stored for long periods. (ii) The preamplifier probe shown in Figure 3 hybridizes to the 28-base template formed by the tandem Z-probes. Specificity is increased since the preamplifier will not bind unless two Z-probes are bound in tandem to the target transcript, thus eliminating background signal from Z-probes that bind nonspecifically (hence not in tandem). (iii) The preamplifier probe contains as many as 20 common binding sequences for the next stage involving the amplifiers. (iv) Each amplifier probe contains as many as 20 common sequences, which act as templates for the binding of the labeled detector oligonucleotide probes. Overall, RNAscope can achieve a theoretical maximum of 8000-fold (20 × 20 × 20) signal amplification from a single transcript and has been used for example to detect single transcript molecules in individual cells.^52^ However, RNAscope is currently limited to reading out only 4 probes simultaneously due to overlap of excitation and emission bands of the different fluorophores used, thus necessitating the use of cycling techniques to achieve higher multiplicity. RNAscope can be used to target any mRNA transcript since the bottom of the Z-probe can be varied to determine target specificity. Twelve different tail sequences (top of the Z-probe), referred to as T1-T12, are commercially available for ACD’s “HiPlex” assay format. For this reason, we kept our MALDI-ISH experiments within this range for proof-of-concept. In principle, much higher multiplexing should be possible with MALDI-ISH (see Conclusion).

**Figure 3:**
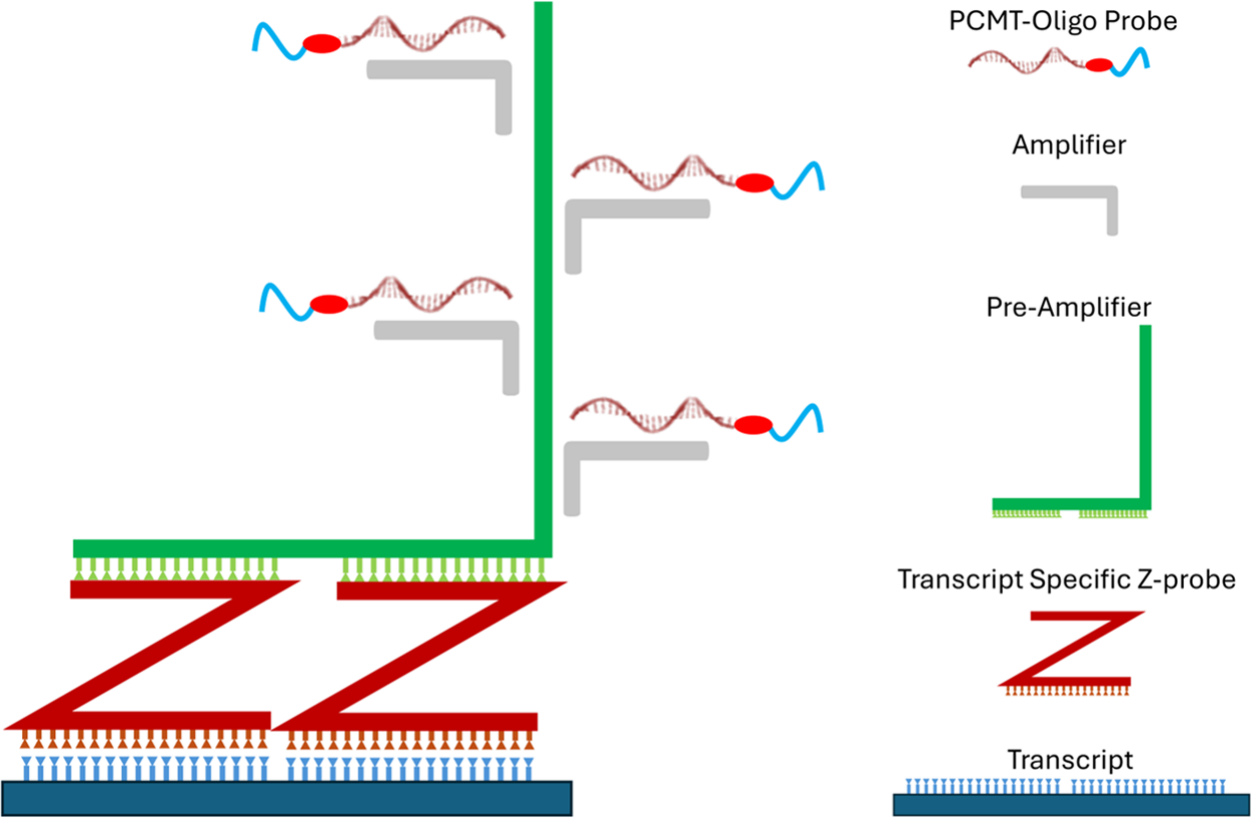
Diagram of the RNAscope branched DNA (bDNA) method used for amplified MALDI-ISH. Pairs of custom Z-probes (dark red Z shapes) target adjacent transcript sequences (dark blue) to form a 28-bp template for binding of the preamplifier probe (green). As many as 20 Z-probes are utilized to recognize different sequences of the same target transcript. The preamplifiers contain up to 20 identical template sequences for binding of the amplifier probes (gray). The amplifiers also contain 20 template sequences for the PCMT-oligonucleotide probes to bind. Overall, this approach can produce a theoretical maximum of 8000-fold amplification.

The HiPlex RNAscope assay was performed essentially according to the manufacturer’s instructions for fresh frozen (FF) tissue sections, except PCMT-oligonucleotides were used instead of fluorescent oligonucleotides at the detector probe step, and the final washes were modified to remove salts that are incompatible with MALDI-MSI. hAbeta^SAA^ (APP-SAA) transgenic Alzheimer’s disease (AD) and wild-type (WT) mouse brain FF tissue sections were used as the samples, and the 10 μm cryosections were mounted on Bruker IntelliSlides. MALDI-MSI was performed with a timsTOF fleX MALDI-2 system (Bruker Daltonics, Billerica, MA) in reflector mode with 20 μm spatial resolution. While the timsTOF flex MALDI-2 can achieve 5 μm spatial imaging, 20 μm resolution was chosen for this study to balance acquisition time and sensitivity. Image and spectral analyses were performed using flexImaging, flexAnalysis, and SCiLS Lab software (Bruker Daltonics, Billerica, MA) as well as in-house developed image analysis software based on Python and Fiji/ImageJ.^53^

Figure 4a–c shows overlaid multicolor spatial maps of the relative intensities of 7 of the 10 mass reporters photoreleased from the PCMT-oligonucleotide probes (referred to as MALDI-ISH images) from a 10-plex experiment. Distinct and specific patterns are observed when Z-probes were used that target specific mouse brain transcripts (Figure 4a is WT and Figure 4b is the AD mouse brain; histology of the observed transcript patterns is discussed later). Note that with the color-blending occurring in Figure 4a and b due to the 7-color overlay and transcript colocalization in some cases, it can be difficult to discern the patterns of individual transcripts; therefore, single-ion images are shown in Supplementary Figure S1 for all 10 targeted transcripts. Table 1 (see Methods section) lists all targeted transcripts as well as tail and PCMT assignments. Importantly, Figure 4c, the negative control, shows no significant signal or specific patterns, as expected. The Z-probes used for the negative control contain the same tails (upper portion of a Z-probe), and the tissue section (WT in this case) was subjected to the full MALDI-ISH procedure, including using the same PCMT-oligonucleotide detector probes for readout. However, the Z-probes were targeted to bacterial transcripts absent from the mouse brain. Figure 4d shows the overall average spectrum from the WT tissue section shown in Figure 4a, with the mass reporter peaks indicated by the transcript (gene) names (and small blue arrowheads on the *x*-axis). The mass reporters are the dominant peaks with relatively minor contaminating molecular species, which are either endogenous biomolecules from the tissue or MALDI-ISH buffer constituents which remain on the slides in trace amounts, similar to previously observed with the MALDI-IHC antibody-based approach.^33^

**Figure 4:**
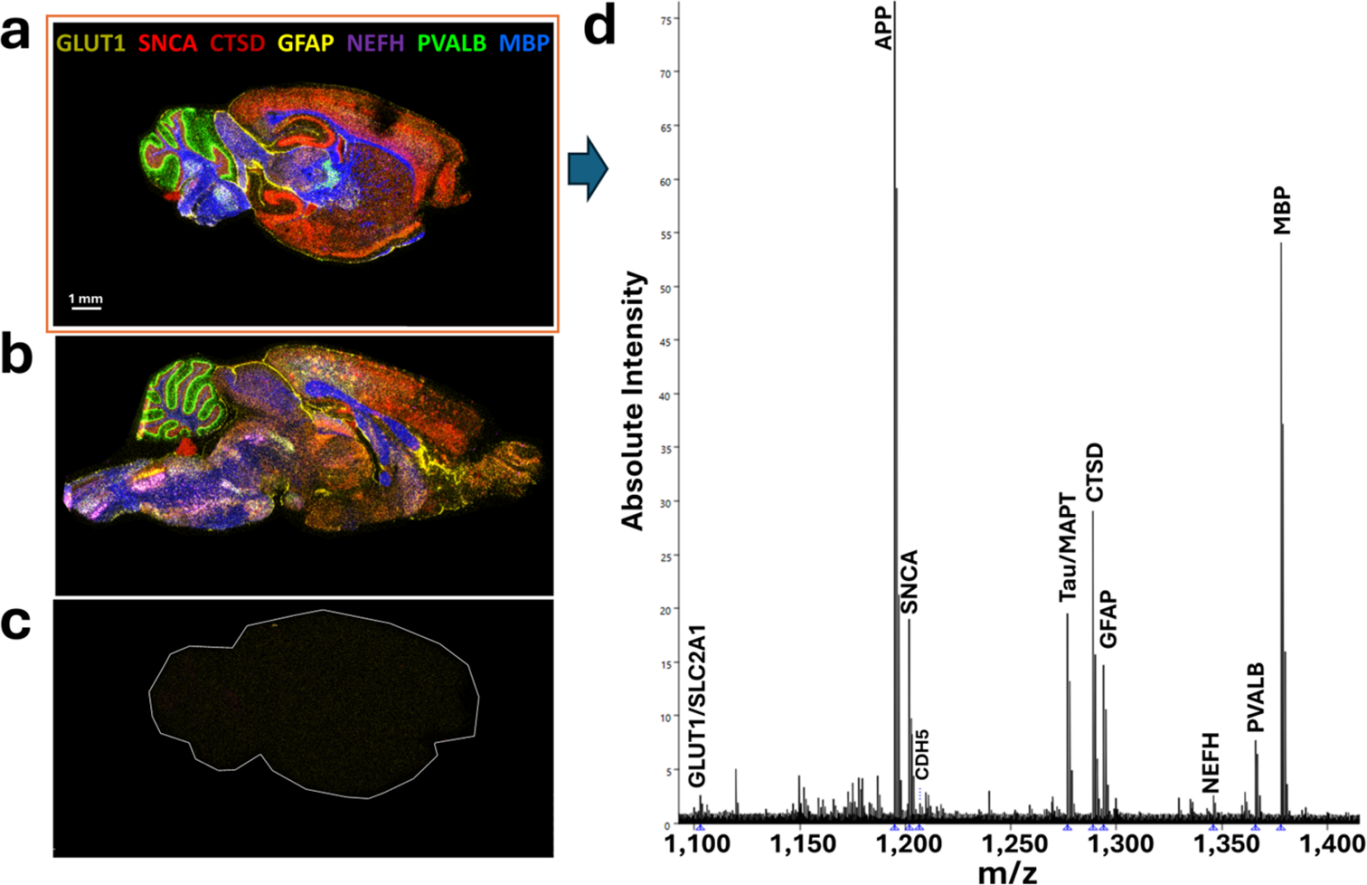
Amplified 10-plex MALDI-ISH imaging of wild-type (WT) and Alzheimer’s disease (AD) transgenic hAbeta^SAA^ (APP-SAA) fresh frozen (FF) sagittal mouse brain tissue sections. (a–c) Peak intensities of the PCMT mass reporters were used to construct MALDI-ISH images of the targeted transcripts. (a, b) WT and AD transgenic hAbeta^SAA^ (APP-SAA) mouse brain tissue sections, respectively, were subjected to the 10-plex MALDI-ISH procedure whereby the transcript-specific Z-probes targeted specific mouse brain transcripts, with multicolor overlaid images shown for 7 of the transcripts according to the color key provided (GLUT1, gold; SNCA [α-synuclein], red; CTSD [cathepsin D], dark red; GFAP [glial fibrillary acid protein], yellow; NEFH [neurofilament heavy chain], purple; PVALB [parvalbumin], green; MBP [myelin basic protein], blue). (c) Negative control. WT mouse brain was subjected to the full 10-plex MALDI-ISH procedure, including the same Z-probe tails, amplifiers, and PCMT-oligonucleotide detector probes as in panels a and b, except the transcript-specific Z-probes targeted bacterial sequences absent from mouse brain. The overlaid multicolor negative control image was constructed from the same mass reporters from the same PCMT-oligonucleotide detector probes as in panels a and b. MALDI-ISH Image Display Settings: Using Bruker’s SCiLS Lab Version 2025b Pro software, the lower display threshold was set to 10% and the upper display threshold was set to 100%, applied uniformly to all analytes and all tissue sections. (d) Overall average spectrum from the entire WT tissue section shown in panel a, with the PCMT mass reporter peaks labeled with the corresponding transcript names. All tissue sections were fresh frozen. All MALDI-ISH imaging was performed at a 20 μm spatial resolution.

For a basic quantification of the staining specificity of the probes, the signal-to-background ratio was calculated for each of the 10 transcripts from the WT tissue section in Figure 4a using the negative control in Figure 4c as the background. Since transcripts can be locally abundant but not necessarily uniformly distributed over the entire tissue section, to measure the “signal”, mean PCMT intensities were calculated from subregions of the tissue in Figure 4a that were positive for each of the 10 transcripts (see small green circles in the single ion images in Supplementary Figure S1). Note since the negative control was uniform, the mean intensity of the same PCMTs from the entire tissue section in Figure 4c was taken as the background value. Signal-to-background ratios ranged from 3 to 53:1 (PVALB 34:1, GFAP 28:1, GLUT1/SLC2A1 3:1, APP 37:1, SNCA 31:1, CTSD 39:1, NEFH 13:1, MBP 53:1, Tau/MAPT 13:1, and CDH5 6:1).

In addition to the clean negative control and specific and distinct transcript patterns shown in Figure 4, we sought to further validate these results by comparison to the published literature and databases reporting the histology for specific transcripts obtained using conventional methods. Figure 5a shows a comparisons between MALDI-ISH and conventional chromogenic ISH images from the Allen Brain Atlas^54–57^ for parvalbumin (PVALB), glial fibrillary acidic protein (GFAP), and amyloid precursor protein (APP) for a WT mouse brain. Considering that different methods and different animals were used for our data versus the Allen Brain Atlas, strong agreement in staining patterns is still clearly observed. To further validate the MALDI-ISH results, serial tissue sections for the AD mouse brain were subjected to conventional fluorescence-based RNAscope.^32^ Fluorescence results shown in Figure 5b for three example transcripts, myelin basic protein (MBP), cathepsin D, and APP, are very similar to the MALDI-ISH results for the same transcripts shown in Figure 5c (notwithstanding the differences in spatial resolution of 0.137 μm/pixel [40× objective] for the fluorescence image and 20 μm/pixel for the MALDI-ISH image; see also later in Figure 6a and b for magnified views for a subregion of the mouse brain).

**Figure 5:**
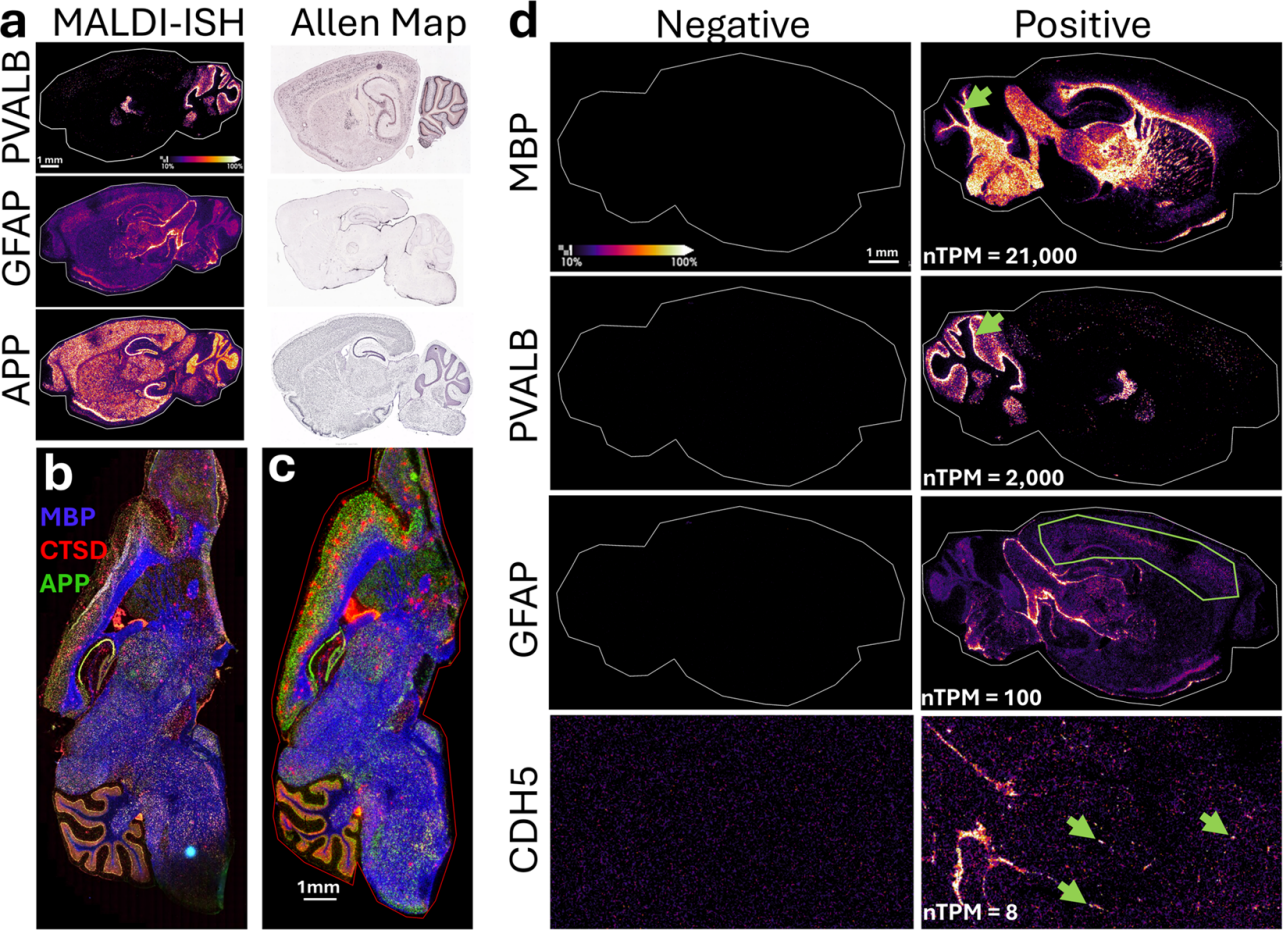
Validation and approximation of the sensitivity and dynamic range of MALDI-ISH by comparison to conventional *in situ* hybridization (ISH) methods and RNA-Seq data. (a) Comparison between MALDI-ISH and conventional chromogenic ISH images from the Allen Brain Map database for wild-type (WT) sagittal mouse brain tissue sections, showing selected transcripts that included PVALB (parvalbumin), GFAP (glial fibrillary acidic protein), and APP (amyloid precursor protein). Allen Map image data sets were as follows: 75457581 PVALB-RP_060523_03_G07-sagittal, Image 6; 79913385 GFAP-RP_071204_04_G06-sagittal, Image 11; and 107 APP-RP_Baylor_253875-sagittal, Image 10. (b, c) Comparison between (b) conventional fluorescence-based RNAscope and (c) MALDI-ISH on Alzheimer’s disease (AD) transgenic hAbeta^SAA^ (APP-SAA) fresh frozen (FF) sagittal mouse brain serial tissue sections for 3 example transcripts, which included MBP (myelin basic protein, blue), CTSD (cathepsin D, red), and APP (amyloid precursor protein, green). (d) Single-ion MALDI-ISH images are shown for selected transcripts spanning a range of abundances in the WT mouse brain. “Positive” denotes tissue sections processed using Z-probes targeting the mouse brain transcripts listed on the left of the images. “Negative” denotes the negative control tissue sections processed using Z-probes targeting bacterial transcripts absent from mouse brain (but with the same Z-probe tails, amplifiers, and PCMT-oligonucleotide detector probes as in the “Positive” tissue sections). “nTMP” values listed in the right column of images are normalized transcript per million values for each transcript obtained from RNA-Seq data from the Human Protein Atlas (which also contains mouse brain data). nTPM data correspond to regional values obtained from 17 dissected subregions of the mouse brain. For example, 21,000 nTPM for MBP (myelin basic protein) in the white matter region (*e.g.*, green arrow), 2000 nTPM for PVALB (parvalbumin) in the cerebellar region (*e.g.*, green arrow), 100 nTPM for GFAP (glial fibrillary acidic protein) in the cerebral cortex (green polygon), and 8 nTPM for CDH5 (Cadherin-5 or VE-Cadherin) throughout the mouse brain (green arrows show representative capillary cross sections and longitudinal sections detected with this marker). MALDI-ISH Image Display Settings: Using Bruker’s SCiLS Lab Version 2025b Pro software, the lower display threshold was set to 10% and the upper display threshold was set to 100%, applied uniformly to all analytes and all tissue sections except in the case of CDH5, where due to the lower signal-to-noise ratio the lower threshold was set to 25% and the upper threshold was to 100% for both the Negative and Positive tissue sections. For panels a and d, the “Thermal” color scheme was used in SCiLS Lab. All tissue sections for MALDI-ISH and conventional fluorescence RNAscope were fresh frozen. All MALDI-ISH imaging was performed at 20 μm spatial resolution, and all microscopy was performed at 0.137 μm/pixel using a 40× objective.

**Figure 6:**
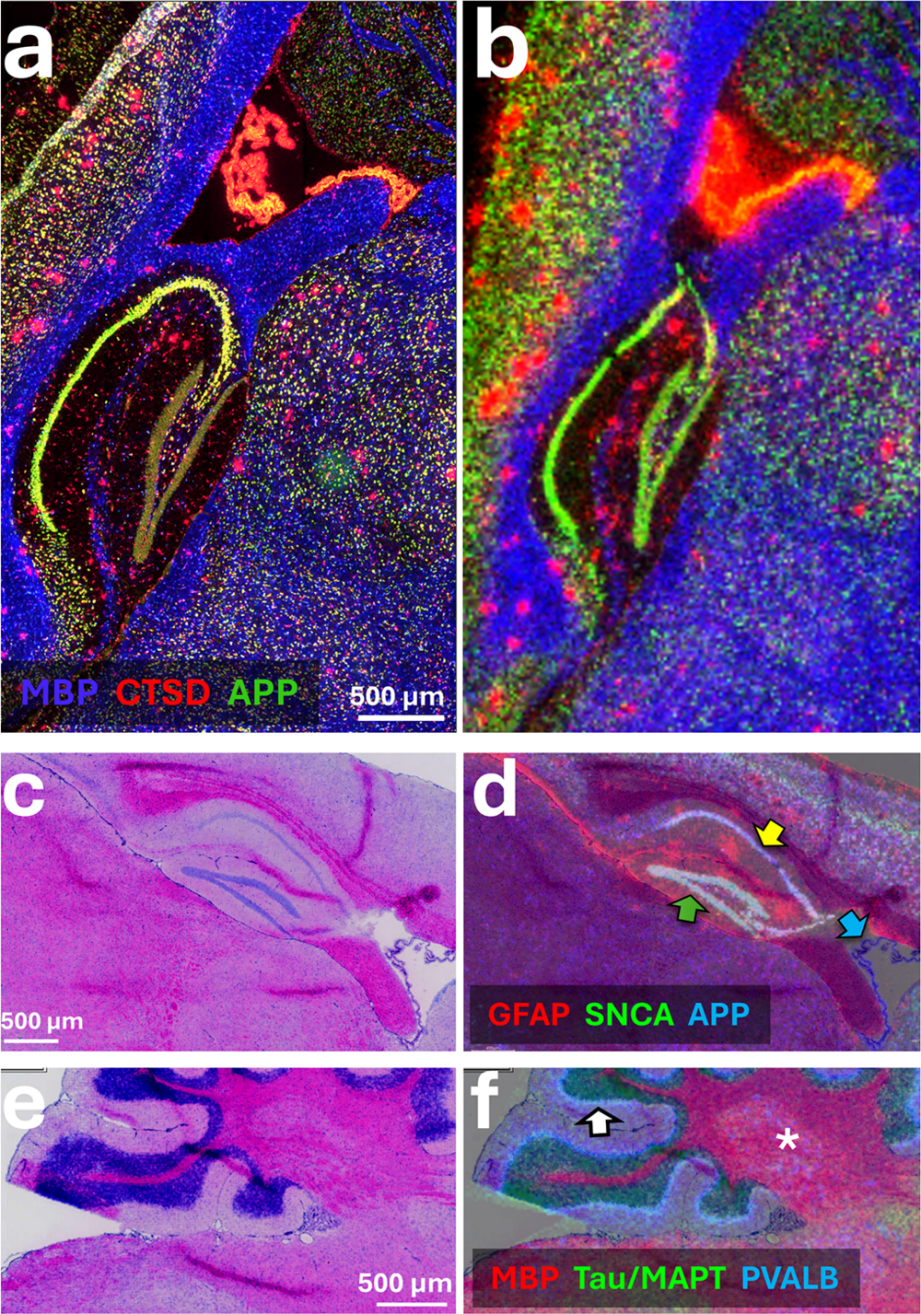
Magnified subregions of sagittal hAbeta^SAA^ Alzheimer’s mouse brain comparing MALDI-ISH to the histology observed by optical microscopy. (a) Magnified subregion of the conventional fluorescence-based RNAscope from Figure 5b compared to (b) the MALDI-ISH from Figure 5c for the same magnified subregion (serial tissue sections for fluorescence and MALDI-ISH) for three example transactions that included MBP (myelin basic protein, blue), CTSD (cathepsin D, red), and APP (amyloid precursor protein, green). (c) Post-MALDI-ISH H&E staining and brightfield microscopy of the hippocampal region. (d) H&E from panel c overlaid with MALDI-ISH for three example transcripts (note MALDI-ISH and H&E images were from the same tissue section). The transcripts are GFAP (glial fibrillary acidic protein, red), SNCA (α-synuclein, green), and APP (amyloid precursor protein, blue). Structures include the granule cell layer of the dentate gyrus (green arrow), the pyramidal layer of Cornu Ammonis (CA1–3, yellow arrow), and the choroid plexus of the lateral ventricle (blue arrow). (e) H&E staining image of the cerebellar/medulla region. (f) H&E image from panel e overlaid with MALDI-ISH images for three example transcripts (note MALDI-ISH and H&E images were from the same tissue section). The transcripts are MBP (myelin basic protein, red), MAPT (microtubule associated protein tau, green), and PVALB (parvalbumin, blue). Structures include the white matter region (white asterisk) and the Purkinje cell layer (white arrow). All tissue sections were fresh frozen. All MALDI-ISH imaging was performed at 20 μm spatial resolution, and all microscopy was performed at 0.137 μm/pixel using a 40× objective.

For further validation of the amplified MALDI-ISH and approximation of the sensitivity, comparisons were made to data from the Human Protein Atlas database.^58^ Note that in addition to human protein immunohistochemistry (IHC) data, the Human Protein Atlas also has absolute quantitative spatial transcriptomic data for brain tissue, including mouse brain, achieved by dissecting 17 subregions of the brain and performing RNA-Seq, reported as normalized transcripts per million (nTPM).^59^ Although the Protein Atlas data are for specific brain regions only and therefore are not resolved to the pixel-level transcript density in the tissue, they provide a rough approximation of the sensitivity and dynamic range obtained with MALDI-ISH.

Results shown in Figure 5d demonstrate that MALDI-ISH detects transcripts over roughly a 2500-fold range, from 21,000 to 8 nTPM (note this range could be larger as limits have not necessarily been achieved). Myelin basic protein (MBP), the major axonal sheath protein and the most abundant transcript, is expressed at approximately 21,000 nTPM in the white matter of the brain and easily detected by MALDI-ISH (*e.g.*, inner white matter region of the cerebellum denoted by the green arrow). The Protein Atlas indicates parvalbumin (PVALB) transcripts are strongest in the cerebellum at approximately 2000 nTPM compared to a few hundred nTPM in other regions. This is in agreement with MALDI-ISH, which successfully detects a similar staining pattern (see the green arrow). PVALB is a known marker of Purkinje cells and the molecular layer (interneurons) of the cerebellum.^60,61^ PVALB is also associated with GABAergic fast-spiking inhibitory interneurons, which are crucial for learning and memory and have been implicated in AD.^62,63^ In the brain, the glial fibrillary acid protein (GFAP) is a marker for astrocytes. While expressed throughout the brain, the Human Protein Atlas shows that it is 100 nTPM in the cerebral cortex. GFAP is successfully detected by MALDI-ISH in the cerebral cortex (see green outlined region in Figure 5d). Lastly, VE-cadherin (Cadherin-5/CDH5), which is a vascular endothelial cell marker, is the lowest abundance transcript out of the set of 10, at 8 nTPM. While the signal in the MALDI-ISH average spectrum is relatively low (see Figure 4d, CDH5), it is specifically detected in the MALDI-ISH images in comparison to the negative control (Figure 5d, CDH5), in small local regions that are not expected to contribute significantly to the average spectrum. The observed histology of CDH5 is consistent, as expected, with capillary cross sections and longitudinal sections (see CDH5 in Figure 5d which shows magnified subregions of the mouse brain to highlight these histological features, examples of which are indicated by green arrows).

To further put the MALDI-ISH results in a histological context, Figure 6 compares magnified views of MALDI-ISH to optical microscopy of the same subregions of the hAbeta^SAA^ Alzheimer’s mouse brain. All MALDI-ISH imaging was performed at 20 μm spatial resolution, and all microscopy was performed at 0.137 μm/pixel using a 40× objective. Figure 6a and b are from Figures 5b and c for fluorescence-based RNAscope and MALDI-ISH, respectively. Note that as in Figure 5b and c, fluorescence and MALDI-ISH are from serial tissue sections and therefore not necessarily imaging the same cell population within the tissue. Nonetheless, Figure 6a and b again highlights the strong correlation between conventional fluorescence-based ISH and MALDI-ISH. Figure 6c–f compares example transcripts from MALDI-ISH to H&E staining with brightfield microscopy, which in this case was performed on the same tissue section, by matrix removal and conventional H&E staining after MALDI-ISH (see Methods). Figure 6d shows the transcripts for GFAP (glial fibrillary acid protein, red), SNCA (α-synuclein, green), and APP (amyloid precursor protein, blue) overlaid with the H&E for the hippocampal region (see Figure 6c for the standalone H&E image of this region). Here, SNCA and APP transcripts are strong in the granule cell layer of the dentate gyrus (green arrow in Figure 6d) and in the pyramidal layer of the Cornu Ammonis (CA1–3, yellow arrow in Figure 6d), whereas APP is also prominent in the choroid plexus of the lateral ventricle (blue arrow in Figure 6d). Conversely, GFAP is strongest in the glial limitans lining the brain fissures, as expected.^64^ Note these are all regions rich in cell nuclei as indicated by the blue color in the standalone H&E image in Figure 6c. Figure 6f shows the transcripts for MBP (myelin basic protein, red), MAPT (microtubule associated protein tau, green), and PVALB (parvalbumin, blue) overlaid with the H&E for the cerebellar/medulla region (see Figure 6e for the standalone H&E image of this region). MBP, for example, is found in the white matter region of the cerebellum (white asterisk in Figure 6f), and PVALB is found primarily in the Purkinje cell layer (white arrow in Figure 6f) as expected.^62,63^

Finally, while this work focused on FF tissues, it is highly desirable to be able to perform MALDI-ISH on FFPE tissues. For example, FFPE is important for clinical applications, where most tissue specimens are in this format. Furthermore, most tissue archives for various diseases are in the form of FFPE specimens. However, mRNA is typically more degraded in FFPE tissues, with only fragments of the original transcripts present.^65^ bDNA-based methods such as RNAscope are designed to compensate for degraded mRNA by using Z-probes that can recognize short transcript fragments in addition to the amplification.^32^ In order to evaluate this with MALDI-ISH, we again used the hAbeta^SAA^ (APP-SAA) transgenic AD mouse brains but in FFPE format. The protocol again followed standard RNAscope practices, for FFPE tissue in this case, except again the PCMT-oligonucleotide detector probes were again substituted for the fluorescence probes and the final washes were modified to remove incompatible salts. Multicolor overlaid MALDI-ISH images of 5 example transcripts from FFPE AD mouse brain (axial tissue sections in this case) are shown in Supplementary Figure S2 (a and b, for mouse brain-specific Z-probes and negative control bacterial-specific Z-probes, respectively). In total, 8 transcripts were detected, as indicated in the overall average spectrum shown in Supplementary Figure S2c (see also Supplementary Figure S3 for single ion images of all 8 transcripts). Each of the 8 transcripts yielded a specific staining pattern in comparison to the negative control. It should be noted, however, that the signals were generally weaker than the FF tissues and there was some nonspecific oligonucleotide probe binding localized to the cerebellum, as evidenced in the negative control (Supplementary Figure S2b). This background was found to correspond to true PCMT mass spectral peaks, which show the correct isotopic envelope, and not spectral noise (for example, see Supplementary Figure S2c, inset spectrum). Therefore, this represents nonspecific binding of any of the oligonucleotide probes in the amplified hybridization procedure, which could ultimately result in tissue-bound PCMT-probe. In the future, further optimizations of probe concentration and hybridization stringency, as well as blocking and washing conditions, may address this issue.

### A Multiomic Workflow for MALDI-ISH Imaging of Lipids and Transcripts on the Same Tissue Section

A multiomic workflow designed to image label-free lipids and targeted transcripts from the same FF tissue section (hAbeta^SAA^ mouse brain) was developed. This first involved direct MALDI-MSI of untargeted, endogenous, label-free lipids in negative ion mode with a sublimated 1,5-diaminonaphthalene (DAN) matrix. Lipids were tentatively identified on the basis of the LIPID MAPS Structure Database (LMSD) (see Methods).^34–37^ Tentative assignment results from LMSD for all lipids analyzed in this study were made on the basis of searching [M – H]^−^ molecular ion species with a 0.01 *m*/*z* mass tolerance (Supplementary Table S2, tentative assignments presented in sum composition format). Direct lipid imaging was followed by matrix removal and implementation of the RNAscope-based MALDI-ISH protocol. Lipid and MALDI-ISH measurements were made on a Bruker timsTOF fleX instrument at 20 μm spatial resolution.

In order to evaluate whether prior MALDI-MSI of untargeted lipids alters the results of the subsequent MALDI-ISH, comparisons were made between the multiomic workflow and MALDI-ISH alone using hAbeta^SAA^ (APP-SAA) transgenic mouse brains. Using Bruker’s SCiLS Lab software, which is designed for viewing and biostatistical analysis of MALDI-MSI data (Bruker Daltonics, Billerica, MA), the MALDI-ISH data sets from the two independent experimental runs (multiomic vs MALDI-ISH only) were combined into one file and then normalized together using the provided root-mean-square (RMS) algorithm. Overlaid multicolor images of four example transcripts (of 10 total) are shown in Figure 7a for the multiomic approach and Figure 7b for MALDI-ISH only. Notwithstanding some minor inherent differences in histology between the two different tissue sections (*e.g.*, in the olfactory bulb), the images are highly comparable, showing no significant impact of prior lipid imaging on the MALDI-ISH results. To verify this, Figure 7c shows the mean intensity of all 10 PCMT reporter ions corresponding to all 10 transcripts in the experiment, which are again highly comparable between the multiomic approach and MALDI-ISH alone. The one exception is myelin basic protein (MBP), which is nearly 2-fold stronger in the multiomic experiment, yet the correct histology is observed in both cases, such as detection of the white matter of the cerebellum and the Corpus Callosum (Supplementary Figure S4). One possibility is that the lipid MALDI-MSI enhances the target retrieval of the MBP transcripts, yielding higher signals.

**Figure 7:**
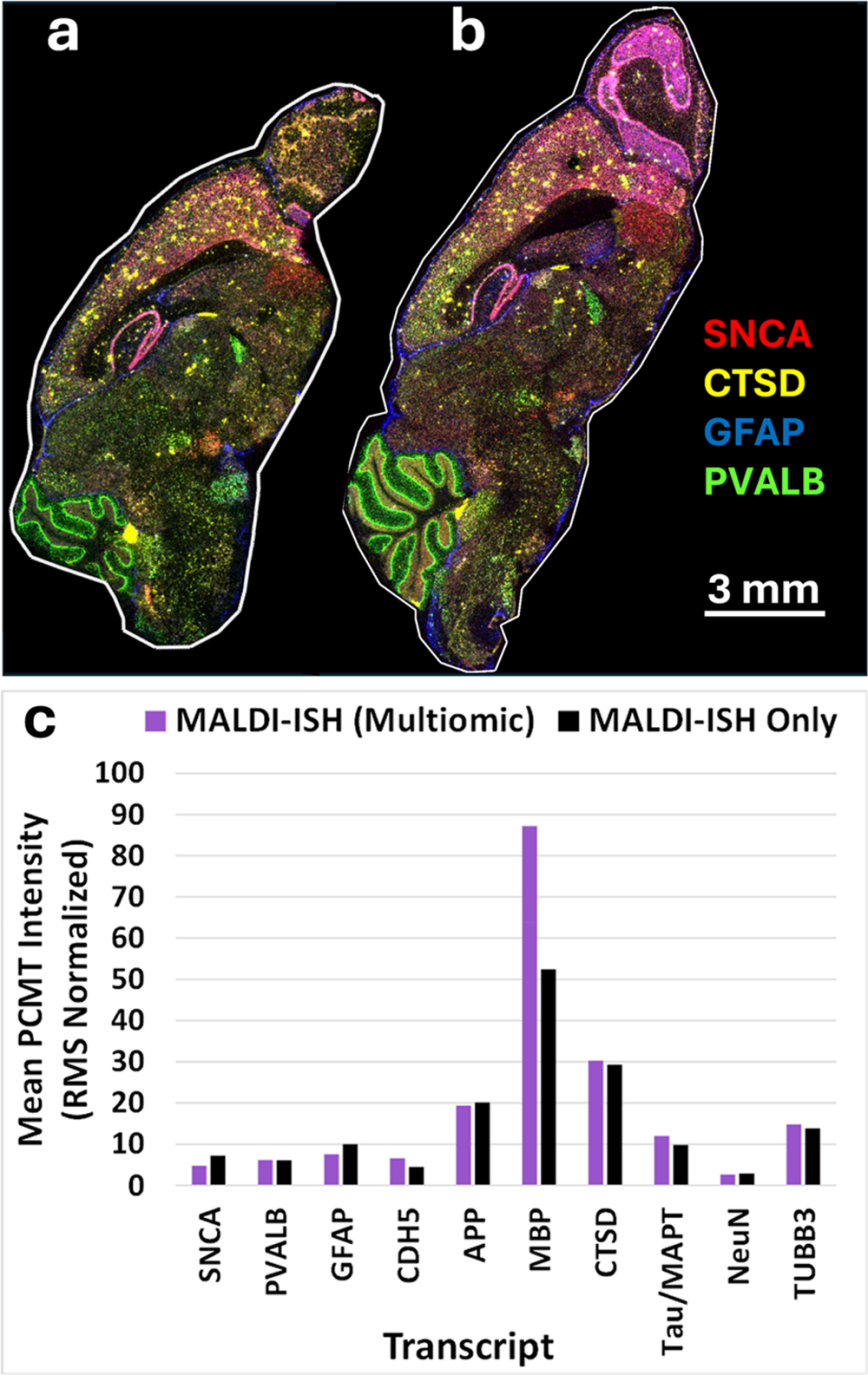
MALDI-ISH which was preceded by untargeted lipid MALDI-MSI on the same tissue section versus MALDI-ISH alone. (a, b) Individual sets of MALDI-ISH images of fresh frozen (FF) Alzheimer’s disease (AD) transgenic hAbeta^SAA^ (APP-SAA) mouse brain sagittal tissue sections. Four example transcripts of the 10 detected in this experiment are shown as indicated by the color key provided; these are SNCA (α-synuclein), red; CTSD (Cathepsin D), yellow; GFAP (glial fibrillary acidic protein), blue; and PVALB (parvalbumin), green. MALDI-ISH Image Display Settings: Using Bruker’s SCiLS Lab Version 2025b Pro software, the lower display threshold was set to 10% and the upper display threshold was set to 100%, applied uniformly to all analytes and all tissue sections. (a) MALDI-ISH was preceded by MALDI-MSI of untargeted, label-free lipids (lipids not shown; see Figure 8 for lipid images from this tissue section). (b) MALDI-ISH which was not preceded by MALDI-MSI of untargeted, label-free lipids (*i.e.*, only MALDI-ISH was performed on this tissue section). (c) Mean intensity (root-mean-square [RMS] normalized) over the entirety of each tissue section of all 10 PCMTs corresponding to all 10 transcript probes detected by MALDI-ISH. “Multiomic” refers to MALDI-ISH that was preceded by MALDI-MSI of untargeted, label-free lipids. All tissue sections were fresh frozen. All MALDI-ISH imaging and MALDI-MSI lipid imaging was performed at 20 μm spatial resolution.

Figure 8a and b presents example multicolored overlaid ion images of the entire AD sagittal mouse brain tissue section for the multiomic approach, showing example analytes from the lipid imaging (Figure 8a) and an image merge between selected lipid and MALDI-ISH analytes (Figure 8b). To create the merged image, the Bruker SCiLS Ion Image Mapper software was used for landmark-based coregistration (Bruker Daltonics, Billerica, MA). Specific spatial patterns for a range of analytes from the lipid and MALDI-ISH modalities were observed following a multiomic K-means clustering analysis and are discussed in more detail below.

**Figure 8:**
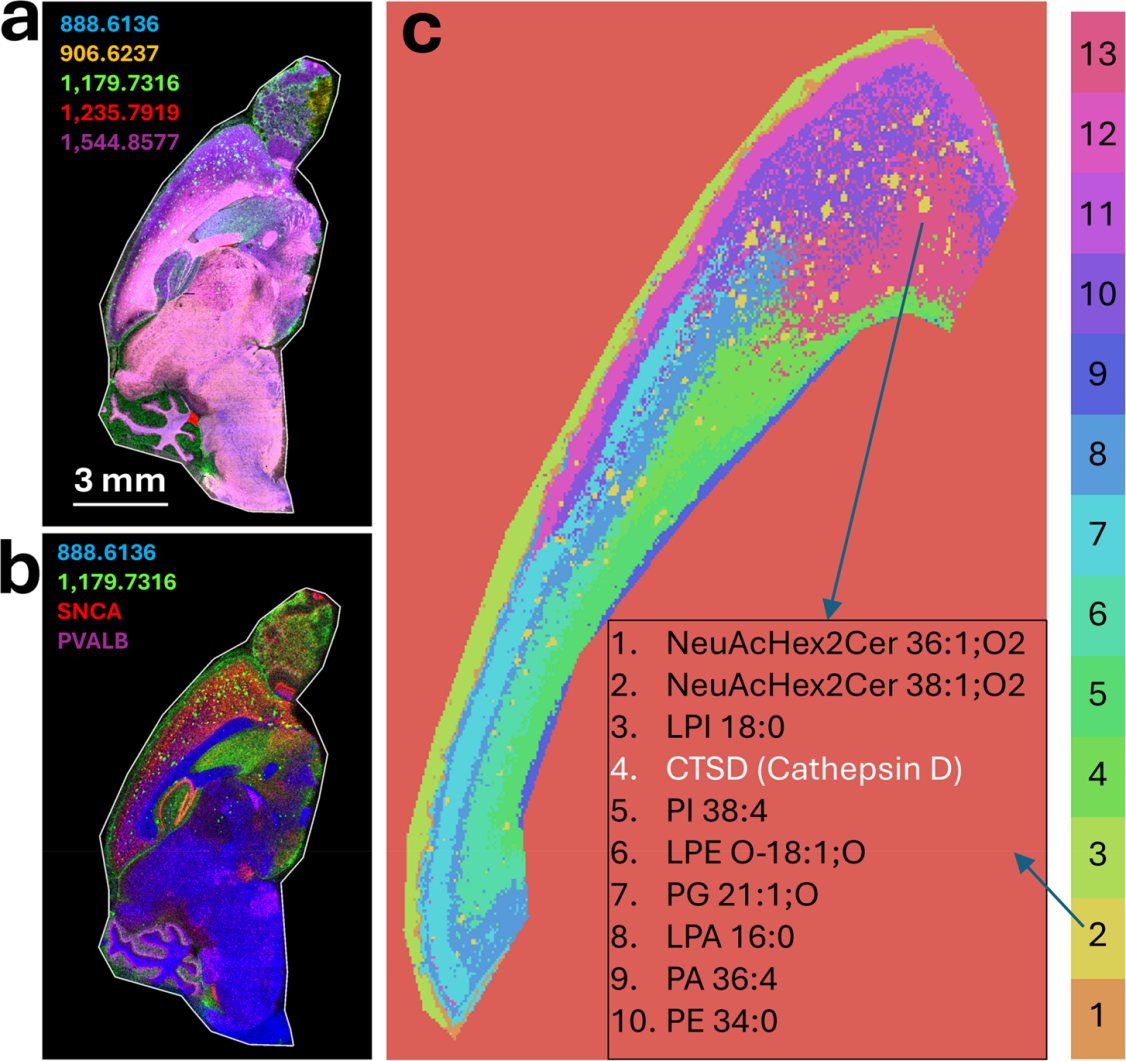
Multiomic MALDI-MSI of untargeted lipids and targeted transcripts (MALDI-ISH) on the same fresh frozen (FF) tissue section. (a, b) Individual sets of images of the same Alzheimer’s disease (AD) transgenic hAbeta^SAA^ (APP-SAA) mouse brain sagittal tissue section imaged successively for lipids and transcripts using a MALDI-based multiomic workflow (same tissue section as in Figure 7a). (a) MALDI-MSI of untargeted, label-free lipids. Example lipids are listed by their observed mass spectral *m*/*z* values, and Supplementary Table S2 provides tentative assignments (in sum composition format) based on the LIPID MAPS database for the >100 lipids used in the analysis. (b) Aligned multiomic image of the same tissue section with two example lipids identified by their observed mass spectral *m*/*z* values (tentatively, sulfatide SHexCer 42:2;O2, blue; and ganglioside GM3 [NeuAcHex2Cer 36:1;O2], green). Example transcripts shown are SNCA (α-synuclein), red, and PVALB (parvalbumin), purple. (c) Multiomic K-means clustering analysis based on >100 lipids and 10 targeted transcripts for the cerebral cortex region of the mouse brain. The 13 clusters detected are color-coded according to the key provided. The text box inset lists the top 10 contributing analytes to the Alzheimer’s amyloid plaque cluster, Cluster 2 (yellow). Lipids are shown in black text, and transcripts are shown in white. Lipid MALDI-MSI and MALDI-ISH Image Display Settings: Using Bruker’s SCiLS Lab Version 2025b Pro software, the lower display threshold was set to 10% and the upper display threshold was set to 100%, applied uniformly to all analytes and all tissue sections. All tissue sections were fresh frozen. All MALDI-ISH imaging and MALDI-MSI lipid imaging was performed at 20 μm spatial resolution.

### Clustering Analyses of the Multiomic Data

While it is not within the scope of this report to perform a biological/biostatistical study of the hAbeta^SAA^ AD mouse brain using the MALDI-ISH-based workflows, some initial image analytics were performed as a proof-of-concept. Toward this end, Figure 8c shows the results of a multiomic K-means clustering analysis, which was performed on the cerebral cortex of the AD mouse brain (from the experiment in Figure 8a and b), using 114 analytes, which included 104 lipids and 10 mRNAs (listed in Supplementary Table S3). Briefly, the biostatistical workflow was conducted using custom Python scripts to prepare the images for analysis, and the clustering was conducted using the Sklearn library (see Methods for details).

The K-means clustering analysis was performed on the cerebral cortex, since this anatomical region is known to be largely affected by the formation of amyloid plaques.^41–44^ Based on the ability to identify an amyloid plaque-based cluster while still maintaining a low number of clusters, 13 was chosen as the optimal number of clusters to analyze the spatial association of the 114 different analytes in this region. Note that the amyloid plaque pattern was confirmed by performing MALDI-IHC on serial sections from the same tissue block using an anti-Aβ42 antibody for the detection of this protein (see later in the Results and Discussion and Supplementary Figure S5 for including MALDI-IHC in the multiomic workflow and in the K-means clustering analysis). The color-coded multiomic cluster map of the cerebral cortex is shown in Figure 8c, with the amyloid plaque cluster (Cluster 2) represented in yellow. The relative contribution of the 114 different analytes to each cluster is shown in the cluster centroid table in Supplementary Table S3.

The identification of the amyloid plaque cluster (Cluster 2, yellow, in Figure 8c) provides some information about its molecular composition in the hAbeta^SAA^ AD mouse brain. One of the major contributors to this cluster is the Cathepsin D transcript (CTSD, see Figure 7a and b for the yellow punctate staining pattern of this transcript). This transcript encodes a soluble lysosomal aspartic endopeptidase that modulates the processing of Amyloid Precursor Protein (APP), being ultimately involved in the secretion of Aβ42 (the major structural feature in amyloid plaques^42,43^ ), the formation of amyloid plaques, and the development of AD.^66–68^ In further corroboration of these observations, the Nixon group has proposed a mechanism for AD that involves faulty neuronal lysosomal acidification, which results in Aβ buildup within enlarged deacidified lysosomes, followed by intracellular formation of Aβ-containing autophagic vacuoles within large membrane blebs. A hallmark of this process, which precedes lysosomal cell death, is the formation of a characteristic PANTHOS structure (named after the poisonous flower). These structures have been associated with cathepsin and microglial/astrocyte invasion.^69,70^ Moreover, the two most dominant lipids in the amyloid plaque cluster are tentatively identified as gangliosides based on the LMSD search (GM3 [NeuAcHex2Cer 36:1;O2] with a *m*/*z* of 1179.7273 and GM3 [NeuAcHex2Cer 38:1;O2] with a *m*/*z* of 1207.7595). Gangliosides are sialic acid-containing glycosphingolipids that are most abundant in the nervous system.^71^ Several studies reported the accumulation of gangliosides in amyloid plaques, with some of them suggesting that the binding of amyloid beta (Aβ) to gangliosides plays an important role in Aβ aggregation.^71–73^ Moreover, Wehrli et al.^74^ and Michno et al.^75^ of the Hanrieder group have performed extensive MALDI-MSI and multimodal lipid imaging in AD mouse models and the human brain and found, for example, amyloid plaque-enrichment of gangliosides GM 1–3 in the post-mortem human AD brain, providing important corroboration of the mouse model results (see also ref 76 for a review).

### Imaging Untargeted Lipids, Targeted Transcripts and Targeted Proteins on the Same Tissue Section

Experiments were conducted to demonstrate the feasibility of combining untargeted lipid imaging, MALDI-ISH of targeted transcripts, and MALDI-IHC of targeted proteins all on the same tissue section. This was achieved in two rounds of MALDI-MSI, one round of untargeted lipid imaging followed by one round to image all PCMTs from MALDI-ISH and MALDI-IHC (*i.e.*, a “one-pot” step in the MALDI-ISH/IHC staining workflow). The lipid imaging followed by MALDI-ISH sample processing was done as before, except that the MALDI-ISH protocol was modified in this case to use a protease-free target retrieval step based on the PretreatPRO reagent commercially available from Bio-Techne/Advanced Cell Diagnostics (ACD) (Newark, CA). The target retrieval step is designed to better expose the transcript sequences by dissociating proteins (see Methods for details). Lack of protease during target retrieval preserves the protein epitopes for subsequent protein detection by MALDI-IHC. Following completion of the MALDI-ISH fluidic processing steps (before slide drying and MALDI-MSI), the slides were treated with a panel of 35 PCMT-antibodies (Supplementary Table S1), followed by MALDI-MSI to image all PCMTs (see Methods for details). Results are shown in Supplementary Figure S5, including a multiomic image overlay of selected analytes from the three biomolecular classes, lipids, transcripts, and proteins. A multiomic K-means clustering analysis of the cerebral cortex region of the hAbeta^SAA^ AD mouse brain on all analytes is shown in Supplementary Figure S5b–d. 180 untargeted lipids were chosen for analysis, which in addition to the 10 targeted transcripts and 35 proteins comprised 225 multiomic analytes across the three biomolecular classes.

The top K-means clustering lipids and MALDI-ISH hits in the amyloid plaque cluster (Supplementary Figure S5d) recapitulate many of the top hits from the prior lipid/MALDI-ISH experiment in Figure 8. This includes the two GM3 gangliosides (NeuAcHex2Cer 36:1;O2 and NeuAcHex2Cer 38:1;O2) and other lipids such as LPI 18:0, PI 38.4, and PG 21:1;O (note, lipid assignments are tentative), as well as the cathepsin D transcript. The MALDI-IHC targeted protein imaging component of this multiomic method adds further information. Even though this multiomic workflow on the same tissue section using the same mass spectrometry instrument has not previously been reported, many of the observed results, such as for the proteins, are corroborated by the literature. Within the top 20 multiomic hits for the amyloid plaque cluster were the following proteins: amyloid-β-42 (Aβ42) which is well-known to be the major structural feature defining the amyloid plaques,^42,43^ APP and nicastrin which is part of the γ-secretase complex responsible for producing Aβ42 from APP;^77,78^ consistent with reports of “immune” invasion of the plaques,^69,70,79–82^ markers of astrocytes (GFAP protein^83^) and microglia (Iba-1 protein^84^); and finally, in addition to cathepsin D (mRNA and protein in this case), and in further support of the aforementioned lysosomal AD hypothesis,^71,72^ Rab7 protein, a small lysosomal GTPase, was also a top hit in the plaques. In further support of these findings, Müller et al. of the Hopf group recently combined lipid MALDI-MSI with MALDI-IHC for a different transgenic AD mouse model, APPPS1,^85^ and there reactive glia, a ganglioside (in that case GM2), and PI 38:4 were found to be colocalized/enriched in the plaques (*e.g.*, see Supplementary Figure S5d where IBA-1 [microglia], GFAP [astrocytes], two GM3 gangliosides, and PI:38:4 were all top hits in the plaque cluster).

Finally, using technical replicates of both wild-type (WT) and hAbeta^SAA^ transgenic AD mouse brain tissue sections (triplicate serial tissue sections for each sample type), initial assessments of MALDI-ISH reproducibility were conducted within this multiomic workflow. Supplementary Figure S6a shows the triplicate MALDI-ISH images for three example transcripts (PVALB, APP, and MBP) for both the WT and AD sample types (*i.e.*, six tissue sections in total). Qualitatively, a high level of reproducibility is observed both in the signal intensities and spatial patterns. The mean PCMT intensities from the entirety of each tissue section for each of 10 transcripts were calculated and averaged among technical replicates of each sample type. Supplementary Figure S6b shows a bar graph of the results, with the error bars representing the standard deviation among the technical replicates. Coefficients of variance (CV) across all 10 transcripts averaged 19% within each sample type. In another basic assessment of reproducibility, the pixel-by-pixel Pearson correlation was calculated for the three technical replicates between the MBP transcript and a lipid, tentatively identified as sulfatide SHexCer 40:1;O3 (*m*/*z* 878.5953). Sulfatides are known to be enriched in the myelin sheath (Schwann cells/oligodendrocytes) of neuronal axons.^86,87^ Pearson’s *R* values were 0.822, 0.805, and 0.769 for the WT technical replicates and 0.832, 0.821, and 0.775 for the AD technical replicates, for CVs of 3% and 4% for each sample type, respectively (see Supplementary Table S5 for more analyte comparisons).

## Conclusions

We demonstrate a new method for imaging transcripts in tissues based on MALDI-MSI and the design of novel PCMT-oligonucleotide probes that are substituted for fluorescence oligonucleotide probes in conjunction with RNAscope, a form of bDNA amplification.^32^ Furthermore, we demonstrate for the first time a “tri-omic” workflow, which enables label-free metabolites such as lipids as well as targeted transcripts and proteins to be imaged, all from the same tissue specimen on the same mass spectrometry platform. This involved combining established MALDI-IHC methods with MALDI-ISH, enabling imaging of label-free metabolites, RNA and proteins on the same tissue section, with only two MALDI-MSI imaging scans. These approaches facilitated the application of image analysis methods such as multiomic K-means clustering, which can increase biological insight into the complex interaction of different species of molecules in a spatial context. As an initial example, these workflows and analyses were performed on transgenic AD mouse brain tissue to reveal the spatial correlation between different analytes, including label-free lipids as well as targeted transcripts and proteins, which agree with earlier studies (see Results and Discussion).

Importantly, since the PCMT-oligonucleotides comprise the detector probes used as the last hybridization step, they can be adapted to other amplified or unamplified multiplex ISH methodologies. Examples include SABER-FISH,^88,89^ which uses the primer exchange reaction (PER) to generate long nucleic acid concatemer probes which contain a transcript-specific hybridization sequence plus many repeating sequences for binding of labeled detector oligonucleotides; Xenium from 10× Genomics (Pleasanton, CA), which uses circularization of padlock probes bound to the target transcript, followed by rolling circle amplification to create binding sites for multiple labeled detector oligonucleotides;^90,91^ HCR RNA-FISH from Molecular Instruments (Los Angeles, CA), which uses labeled metastable hairpin oligonucleotide probes, whereby binding to a target sequence (initiator) results in strand displacement and hairpin opening, which triggers an *in situ* polymerization reaction of the labeled probes at the target sequence^92^ (referred to as the hybridization chain reaction or HCR); and Thermo Scientific’s (Waltham, MA) ViewRNA, which uses a form of bDNA amplification^93^ similar to RNAscope. Direct probe hybridization to target RNA without amplification is also possible using conventional FISH and its derivatives. For example, MERFISH^94^ (multiplexed error-robust fluorescence *in situ* hybridization) uses the direct hybridization in conjunction with a cycling, single-molecule resolution, fluorescence barcoding scheme, although bDNA amplification has also been applied.^95^ However, all fluorescence-based optical imaging methods require some type of cycling to achieve a high level of multiplexing, which can limit the throughput. In contrast, PCMT-conjugated detector oligonucleotides could be substituted for the fluorescence oligonucleotides and imaging/decoding achieved without cycling using MALDI-MSI. In the case of direct hybridization-based approaches, transcript-specific probes carrying more than one PCMT are also possible to increase signal without prior amplification, for example, by using overhang sequences that are not part of the transcript-binding region but carry many PCMTs.

Although our work here was limited to 10 targeted transcripts with RNAscope-based MALDI-ISH, much higher multiplexing should be possible since this is not a limitation of the PCMT approach. While 120-plex has already been achieved with the MALDI-IHC antibody-based approach^96,97^ using PCMTs having the same mass reporter structure as MALDI-ISH, we anticipate the limit using a conventional MALDI-TOF instrument in reflector mode will conservatively be 500-plex (*e.g.*, spanning a 2500 *m*/*z* mass window, with a 5 *m*/*z* spacing of different PCMTs to avoid isotopic envelope overlap, and ∼40,000 mass resolution [Mass/fwhm] on a Bruker timsTOF fleX for example). In future work, coupling a second dimension of separation to the MALDI-TOF, such as with trapped ion mobility (TIMS) available on the Bruker timsTOF fleX instrument used here, should increase the multiplexing capabilities by additionally utilizing the collisional cross-section (CCS; *i.e.*, molecular “shape”) of the PCMTs in addition to the *m*/*z* measurement from the subsequent TOF separation. Since there is no cycling involved in MALDI-ISH, scan times are independent of “plexity”, and at the 20 μm spatial resolution used here, this corresponds to an acquisition speed of 4 h/cm^2^.

Potentially the most critical benefit of MALDI-ISH is its multiomics capability on the same tissue section using the same mass spectrometric platform. MALDI-MSI has the unmatched capability to directly image label-free small molecules of nearly any kind, as discussed earlier in the Introduction, making it an ideal platform on which to build a multiomic approach. While multimodal approaches have combined other omics platforms such as multiplex IHC or spatial transcriptomics with MALDI-MSI,^98,99^ several challenges exist. This requires multiple different expensive instruments, and the use of different platforms renders image coregistration complex and less accurate, a problem further confounded by differing spatial resolutions. Moreover, rather than the same tissue section, serial sections are sometimes required since the different platforms can require specialized slides (*e.g.*, MALDI-MSI often requires conductive slides and the 10x Genomics Visium platform^100^ requires RNA capture slides). Serial tissue sections may also be required since methods such as imaging mass cytometry^101^ (IMC; a form of multiplex IHC) are destructive and completely ablate the tissue, limiting the order of operations for layered multimodal approaches, which can be suboptimal. Using serial tissue sections can also be problematic since they are not identical and can comprise different cell populations. In addition, sample specimens, for example, from tissue biopsies, are often precious and in limited supply.

Here, we have greatly expanded the capabilities of conventional MALDI-MSI to include multiplexed targeted transcript imaging using PCMT-conjugated affinity probes. Initial studies also indicate that MALDI-IHC can be added to the multiomic workflow for targeted protein imaging on the same tissue sections using the same mass spectrometry platform (see Results and Discussion and Supplementary Figure S5). Additional types of molecular species can also be included by using additional MALDI-MSI imaging steps. This includes MALDI-MSI of protein post-translational modifications, such as specific glycosylations using PCMT-lectin probes,^27^ or by utilizing glycosidases to remove and directly image the liberated glycans,^11^ as well as other MALDI-MSI imaging modalities.^102^ MALDI-MSI of extracellular matrix peptides, for example, using *in situ* collagenase digestion, can also be performed in conjunction with targeted PCMT-probes.^11^ It is also noted that unlike imaging mass cytometry (IMC), MALDI-ISH and MALDI-IHC can achieve very high multiplexing capability.^96,97^ These advances will potentially provide more powerful methods for researchers to explore the spatial distribution of biomolecules in tissues at the regional and cellular level in various fields including proteomics, tissue pathology, tissue diagnostics, therapeutics, and precision medicine.

## Supplementary Material





## Notes

Single-ion MALDI-ISH images; mass spectra; multiomic MALDI-MSI of untargeted lipids, targeted mRNA transcripts, and targeted proteins on the same fresh frozen tissue sections; and MALDI-ISH reproducibility analysis (PDF)
Tables corresponding to the antibody panel used, tentative lipid assignments in sum composition format, cluster centroid coordinates for multiomic K-means analysis, top analyte hits from multiomic K-means analysis, and analyte colocalization Pearson correlations (XLSX)

## Abbreviations

AD: Alzheimer’s disease
MS: mass spectrometry
MALDI: matrix-assisted laser desorption/ionization
MSI: mass spectrometric imaging
MALDI-MSI: matrix-assisted laser desorption/ionization mass spectrometric imaging
PCMT: photocleavable mass-tag
DAN: 1,5-diaminonaphthalene
CHCA: alpha-cyano-4-hydroxycinnamic acid
FFPE: formalin-fixed paraffin-embedded
FF: fresh frozen
ISH: *in situ* hybridization
MALDI-ISH: matrix-assisted laser desorption/ionization based *in situ* hybridization
IHC: immunohistochemistry
MALDI-IHC: matrix-assisted laser desorption/ionization based immunohistochemistry
FISH: fluorescence *in situ* hybridization
ESI: electrospray ionization
NHS: *N*-Hydroxysuccinimide
PC: photocleavable
PC-Linker: photocleavable linker
bDNA: branched DNA
LC-MS: liquid chromatography mass spectrometry
Aβ: amyloid-β
Aβ42: amyloid-β-42
PER: primer exchange reaction
HCR: hybridization chain reaction
TIMS: trapped ion mobility
CCS: collisional cross-section
MERFISH: multiplexed error-robust fluorescence *in situ* hybridization
fwhm: full (peak) width at half-maximum
MBG-Water: molecular biology grade water
H&E: hematoxylin and eosin
CV: coefficient of variance
RMS: root mean squared
