# Supplementary material for: Photocleavable Mass-Tagged Oligonucleotide Probes for Multiplexed and Multiomic Tissue Imaging of Targeted Transcripts
Source: J Am Soc Mass Spectrom. 2025 Jul 11;36(8):1621–40. doi: 10.1021/jasms.5c00057 (PMC12333371; doi:10.1021/jasms.5c00057)
Supplement: Supplementary file 1 [file js5c00057_si_001.pdf]

Supporting Information

Photocleavable Mass-Tagged Oligonucleotide Probes for Multiplexed and Multiomic Tissue Imaging of Targeted Transcripts

Jonathan M. Bell[1], Gargey Yagnik[1], Leonardo G. Dettori[1], Philip Carvalho[1], Zhi Wan[1], Kenneth J. Rothschild[1,2*] and Mark J. Lim[1*]

[1]AmberGen, Inc.
44 Manning Road, Billerica, MA 01821

and

[2]Department of Physics and Photonics Center
Boston University
Boston, MA 02215

*Address all correspondence to either Mark J. Lim or Kenneth J. Rothschild, AmberGen, Inc., 44 Manning Road, Billerica, MA 01821, mlim@ambergen.com or krothschild@ambergen.com.

Supplementary Figures

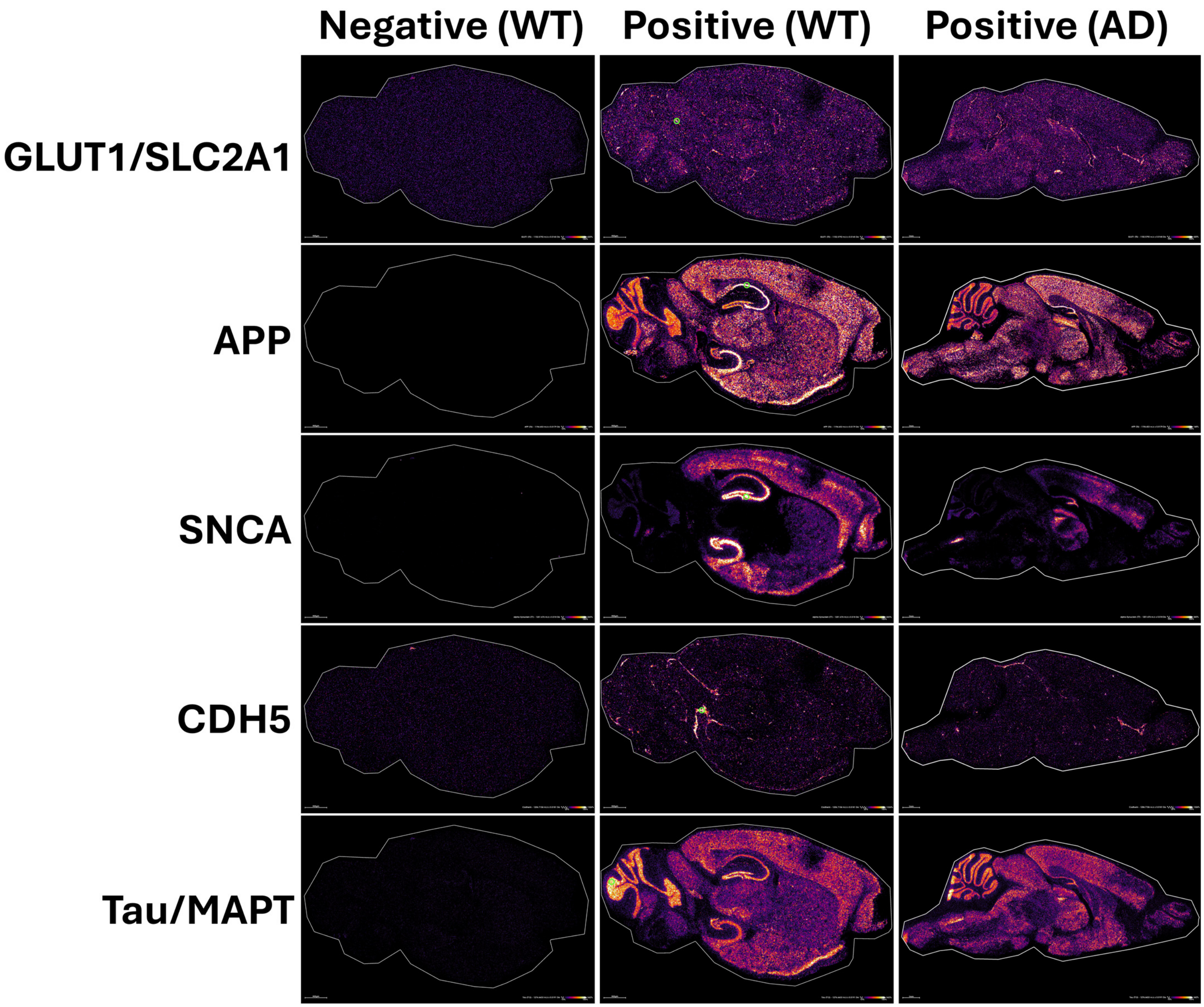

Supplementary Figure S1a. Single ion MALDI-ISH images from the 10-plex experiment on fresh frozen (FF) sagittal mouse brain tissue sections in Figure 4. Supplementary Figure S1a and S1b (next page) show five each of the 10 transcripts. "Positive" denotes tissue sections processed using Z-probes targeting the mouse brain transcripts listed in the figure. "Negative" denotes the negative control tissue sections processed using Z-probes targeting bacterial transcripts absent from mouse brain (but with the same Z-probe tails, amplifiers and PCMT-oligo detector probes as in the "Positive" tissue sections). Wild-type (WT) and Alzheimer's Disease (AD) transgenic hABeta^{SAA} (APP-SAA) mouse brain tissue sections are shown as indicated. MALDI-ISH Image Display Settings: Using Bruker's SCiLS Lab Version 2025b Pro software, the lower display threshold was set to 10% and upper to 100%, applied uniformly to all analytes and all tissue sections; except in the case of CDH5, where due to the lower signal-to-noise, the lower threshold was set to 25% and the upper to 100% for all Negative and Positive tissue sections. "Thermal" color scheme was used in SCiLS Lab. All tissue sections were fresh frozen. All MALDI-ISH imaging was performed at 20 μm spatial resolution.

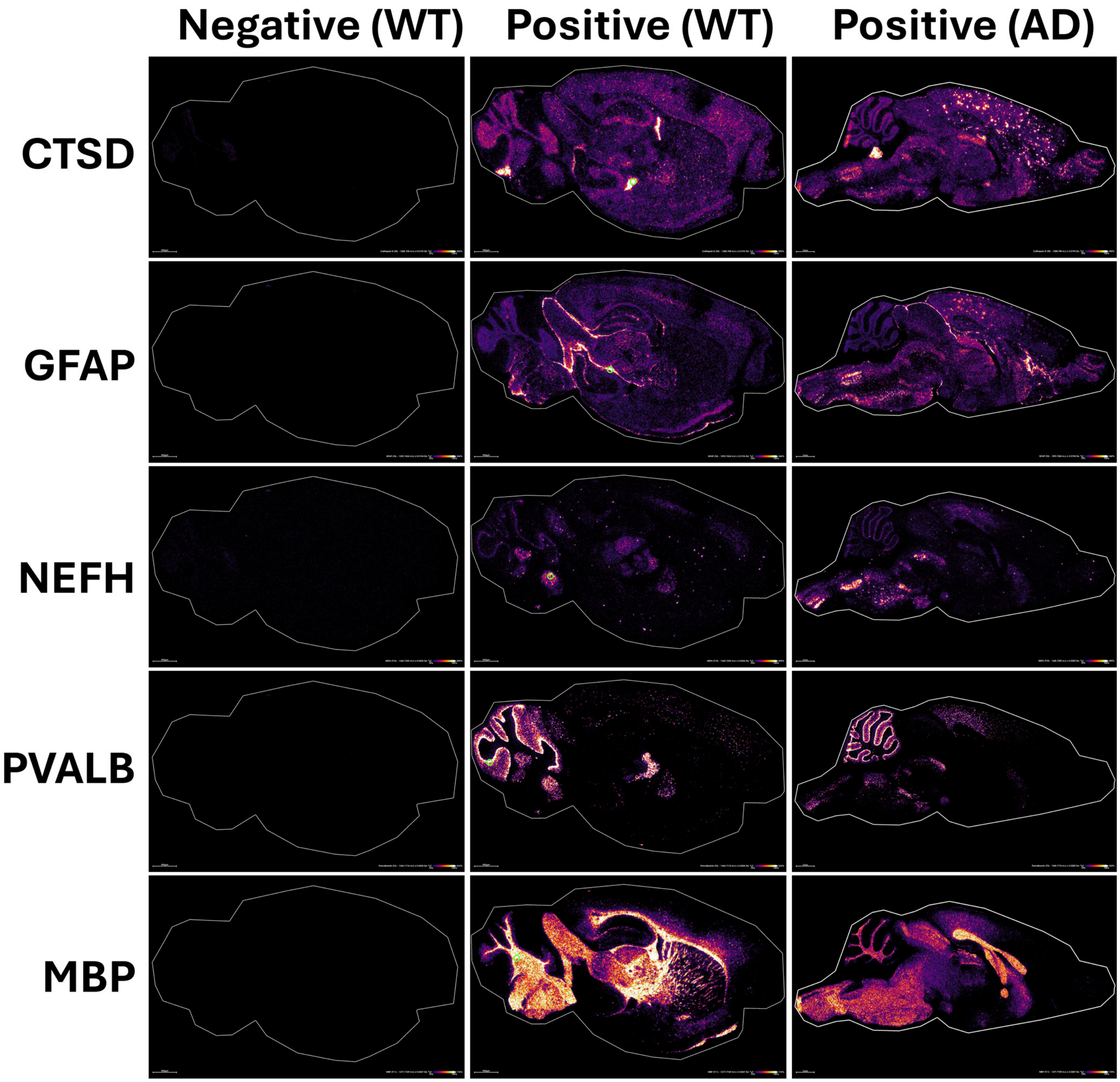

Supplementary Figure S1b. Single ion MALDI-ISH images from the 10-plex experiment on fresh frozen (FF) sagittal mouse brain tissue sections in Figure 4. Supplementary Figure S1a (prior page) and S1b show five each of the 10 transcripts. "Positive" denotes tissue sections processed using Z-probes targeting the mouse brain transcripts listed in the figure. "Negative" denotes the negative control tissue sections processed using Z-probes targeting bacterial transcripts absent from mouse brain (but with the same Z-probe tails, amplifiers and PCMT-oligo detector probes as in the "Positive" tissue sections). Wild-type (WT) and Alzheimer's Disease (AD) transgenic hABeta^{SAA} (APP-SAA) mouse brain tissue sections are shown as indicated. MALDI-ISH Image Display Settings: Using Bruker's SCiLS Lab Version 2025b Pro software, the lower display threshold was set to 10% and upper to 100%, applied uniformly to all analytes and all tissue sections; except in the case of CDH5, where due to the lower signal-to-noise, the lower threshold was set to 25% and the upper to 100% for all Negative and Positive tissue sections. "Thermal" color scheme was used in SCiLS Lab. All tissue sections were fresh frozen. All MALDI-ISH imaging was performed at 20 μm spatial resolution.

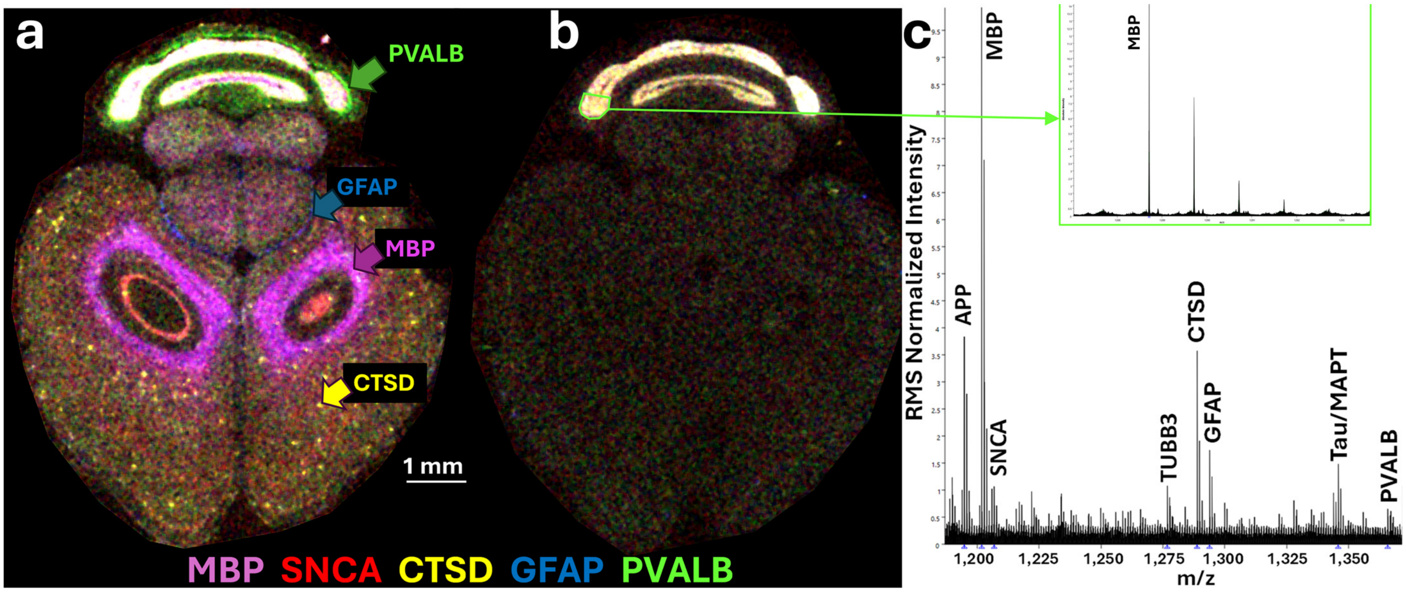

Supplementary Figure S2. Amplified 8-plex MALDI-ISH imaging of Alzheimer's Disease (AD) transgenic hABeta^{SAA} (APP-SAA) formalin-fixed paraffin-embedded (FFPE) axial mouse brain tissue sections. **a-b** Multicolor overlaid images of the 8 PCMT mass reporters used in the MALDI-ISH experiment. **a** Tissue section processed using Z-probes targeting the mouse brain transcripts listed in the figure, per the color key provided. **b** Negative control tissue section processed using Z-probes targeting bacterial transcripts absent from mouse brain (but with the same Z-probe tails, amplifiers and PCMT-oligo detector probes as in panel a). MALDI-ISH Image Display Settings: Using Bruker's SCiLS Lab Version 2025b Pro software, the lower display threshold was set to 10% and upper to 100%, applied uniformly to all analytes and all tissue sections. **c** The main spectrum shown is the overall average spectrum from the entire tissue section shown in panel a, with the PCMT mass reporter peaks labeled with the transcript names. The smaller inset spectrum with the green outline is the PCMT for the MBP probe, averaged from the region of interest outlined in green in panel b (the negative control). All tissue sections were FFPE. All MALDI-ISH imaging was performed at 20 μm spatial resolution.

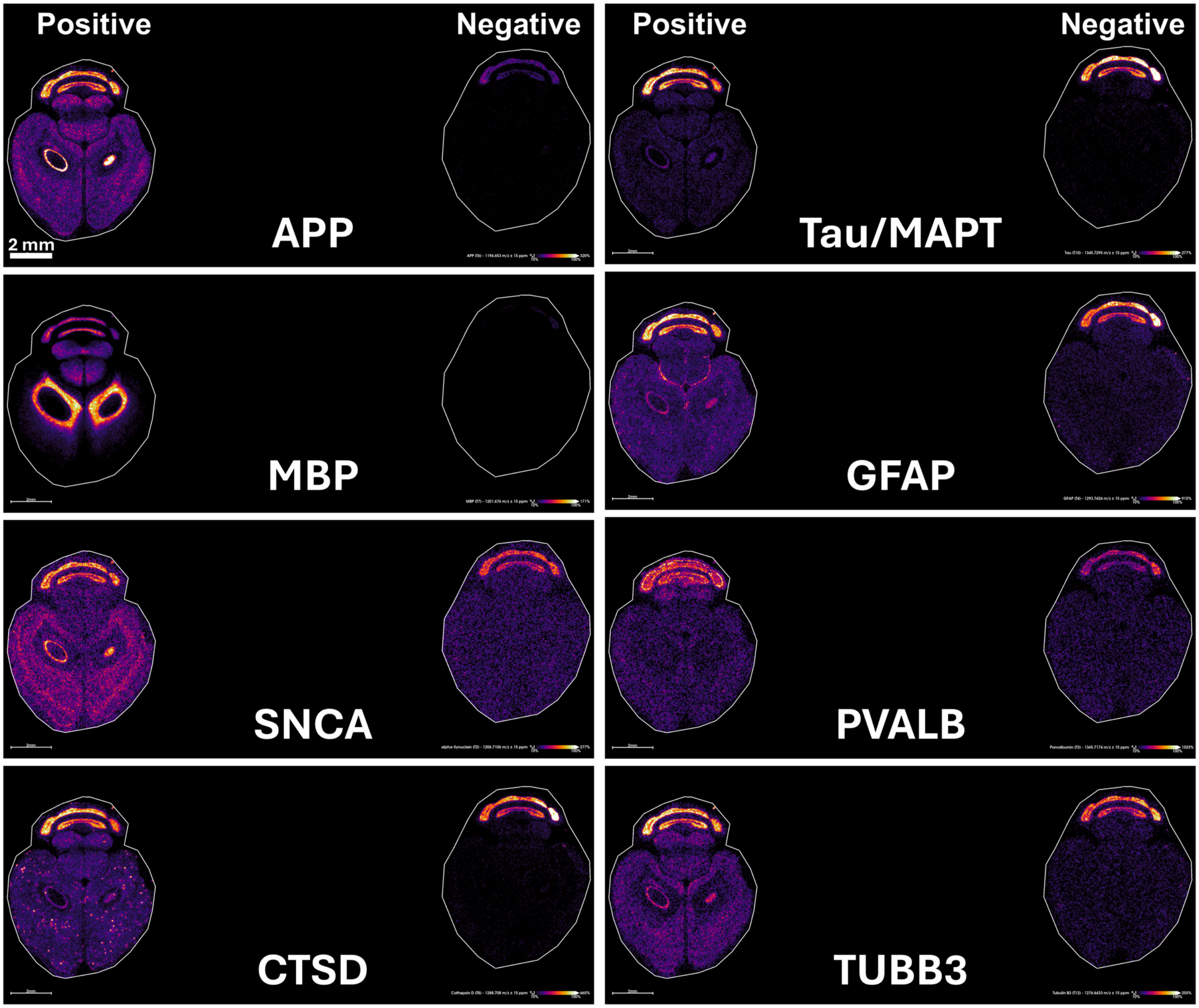

Supplementary Figure S3. Single ion MALDI-ISH images from the 8-plex experiment on formalin-fixed paraffin-embedded (FFPE) mouse brain axial tissue sections in Supplementary Figure S2. “Positive” denotes tissue sections processed using Z-probes targeting the mouse brain transcripts listed in the figure. “Negative” denotes the negative control tissue sections processed using Z-probes targeting bacterial transcripts absent from mouse brain (but with the same Z-probe tails, amplifiers and PCMT-oligo detector probes as in the ”Positive” tissue sections). Alzheimer’s Disease (AD) transgenic hABeta^{SAA} (APP-SAA) mouse brain tissue sections are shown. MALDI-ISH Image Display Settings: Using Bruker’s SCiLS Lab Version 2025b Pro software, the lower display threshold was set to 10% and upper to 100%, applied uniformly to all analytes and all tissue sections. “Thermal” color scheme was used in SCiLS Lab. All tissue sections were FFPE. All MALDI-ISH imaging was performed at 20 µm spatial resolution.

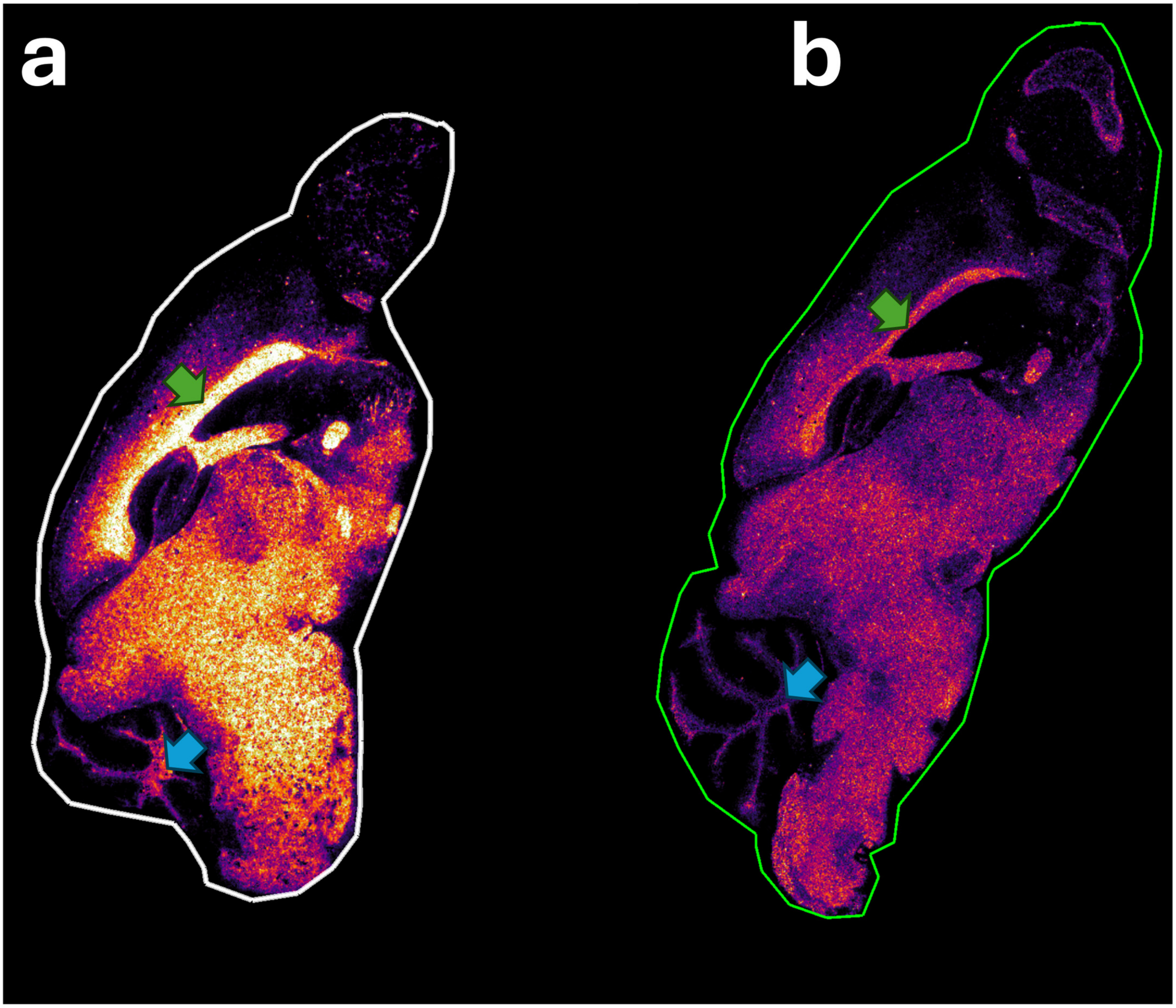

Supplementary Figure S4. Single ion image of MALDI-ISH result for myelin basic protein (MBP) for the experiment from Figure 7 in the main manuscript. **a** MALDI-ISH which was preceded by MALDI-MSI of untargeted, label-free lipids (lipids not shown; see Figure 7 of the main manuscript for lipid images from this tissue section). **b** MALDI-ISH which was not preceded by MALDI-MSI of untargeted, label-free lipids (i.e., only MALDI-ISH was performed on this tissue section). MALDI-ISH Image Display Settings: Using Bruker's SCiLS Lab Version 2025b Pro software, the lower display threshold was set to 10% and upper to 100%, applied uniformly to all analytes and all tissue sections. "Thermal" color scheme was used in SCiLS Lab. All tissue sections were fresh frozen. All MALDI-ISH imaging was performed at 20 µm spatial resolution.

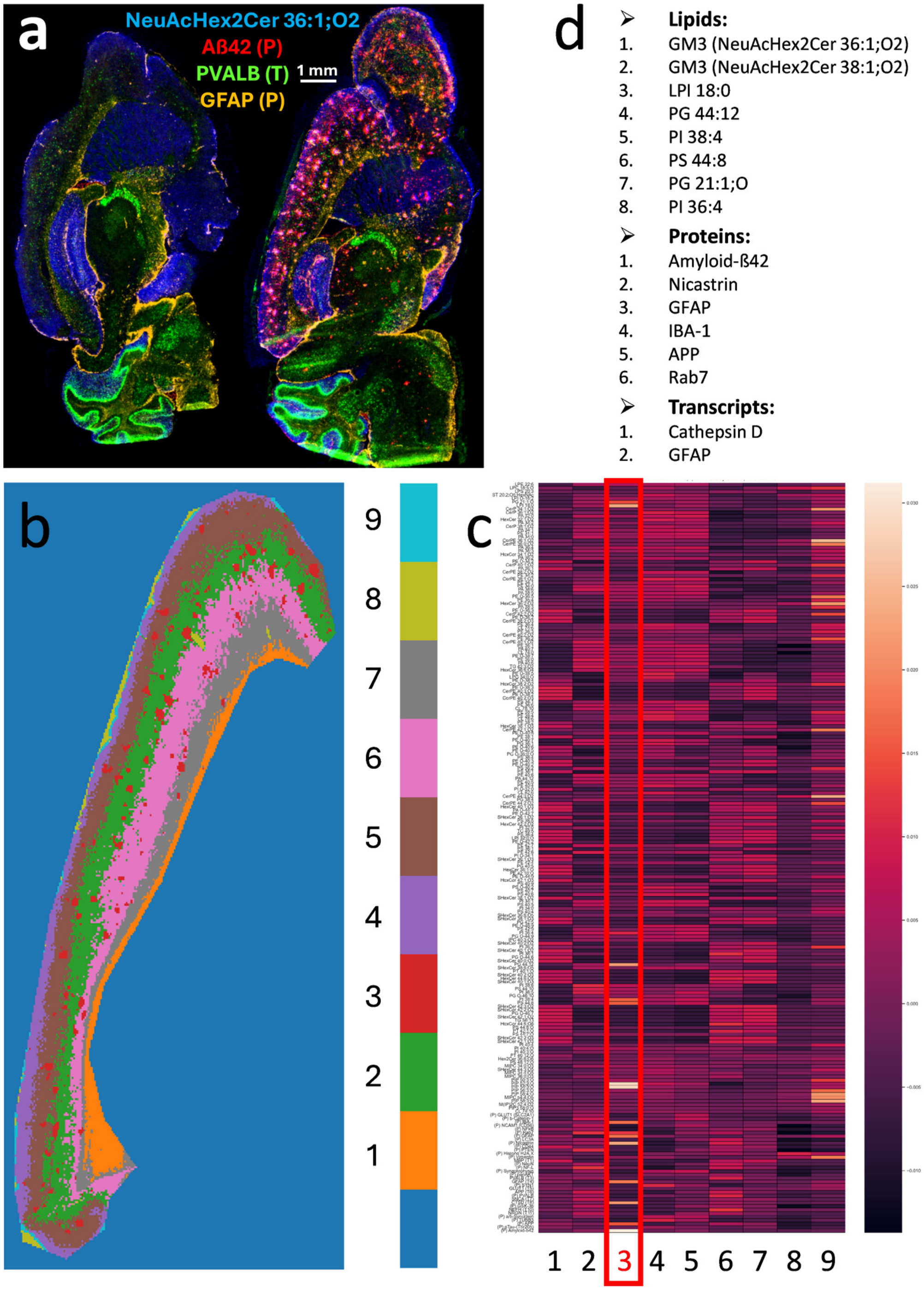

Supplementary Figure S5. Multiomic MALDI-MSI of untargeted lipids, targeted mRNA transcripts (MALDI-ISH) and targeted proteins (MALDI-IHC) on the same fresh frozen (FF) tissue sections. For MALDI-ISH and MALDI-IHC, a panel of 10 oligonucleotide probes (Table 1 of main manuscript) and 35 antibody probes (Supplementary Table S1) was used, respectively, and in addition, 180 untargeted lipids were selected for analysis. **a** Multicolor overlay image of selected analytes from all three biomolecular classes (lipids, transcripts [T] and proteins [P]) for (left) wild-type and (right) Alzheimer's Disease transgenic hABeta^{SAA} (APP-SAA) mouse brain sagittal tissue sections. The example lipid shown (blue) has an m/z of 1,179.7286 and is tentatively the ganglioside GM3 (NeuAcHex2Cer 36:1;O2). The example targeted transcript shown (green) is PVALB (parvalbumin). The two example targeted proteins shown are Aß42 (amyloid beta-42), red; and GFAP (glial fibrillary acid protein), orange. Display Settings: Using Bruker's SCiLS Lab Version 2025b Pro software, the lower display threshold was set to 10% and upper to 100%, applied uniformly to all analytes and all tissue sections. All MALDI-ISH/IHC imaging and MALDI-MSI lipid imaging was performed at 20 µm spatial resolution. **b** Multiomic K-means clustering analysis of all analytes, on the cerebral cortex region of the Alzheimer's Disease transgenic hABeta^{SAA} (APP-SAA) mouse brain. The color bar assigns numbers to each cluster. The amyloid plaques are represented in the red cluster (Cluster 3), forming punctate structures throughout the cerebral cortex. **c** Cluster center coordinates heatmap resulting from the multiomic K-means cluster analysis of the cerebral cortex, including an intensity bar showing relative contribution of each analyte, normalized by cluster. This heatmap illustrates the relative contribution of each of the >200 analytes (listed on vertical axis) to each cluster (listed on horizontal axis), with a lighter color indicating a larger contribution. The amyloid plaque cluster (Cluster 3) is indicated by a red rectangular outline. **d** Listing of key top hits (taken from top 20) contributing to the amyloid plaque cluster (Cluster 3). All tissue sections were fresh frozen. All MALDI-ISH/IHC and lipid imaging was performed at 20 µm spatial resolution.

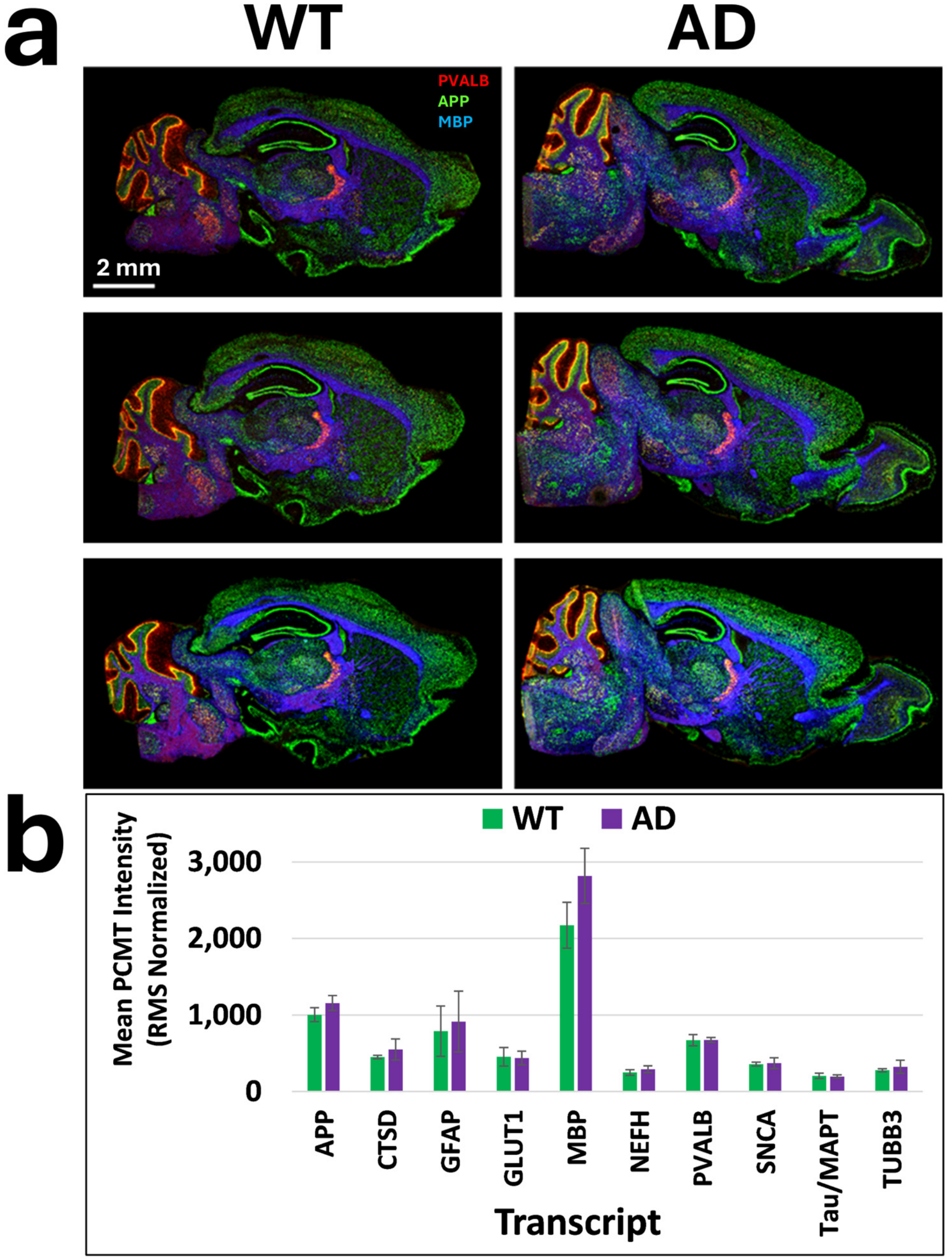

Supplementary Figure S6. MALDI-ISH reproducibility analysis of technical replicates. Data for three technical replicates (i.e., serial sections from same brain block) of each sample type, hAbeta^{SAA} transgenic Alzheimer's Disease (AD) and wild-type (WT) mice, were combined in Bruker's SCiLS Lab software and root mean square (RMS) normalized. **a** MALDI-ISH images of three example transcripts for the triplicate tissue sections of WT (left column) and AD (right column) mouse brain. Transcripts shown are as indicated by the color-key provided in the upper left panel. MALDI-ISH Image Display Settings: Using Bruker's SCiLS Lab Version 2025b Pro software, the lower display threshold was set to 10% and upper to 100%, applied uniformly to all analytes and all tissue sections. All tissue sections were fresh frozen. All MALDI-ISH imaging was performed at 20 µm spatial resolution. **b** Mean PCMT intensities from the entirety of each tissue section were averaged for the triplicate samples and plotted for each of the 10 transcripts, for both the WT (green bars) and AD (purple bars) sample types. Error bars represent the standard deviation of the triplicate samples. %CV across all transcripts averaged 19% within each sample type, AD and WT.
